# A survey of biosecurity practices of pig farmers in selected districts affected by African swine fever in Uganda

**DOI:** 10.3389/fvets.2023.1245754

**Published:** 2023-08-17

**Authors:** John E. Ekakoro, Margaret Nawatti, David F. Singler, Krista Ochoa, Robinah Kizza, Dickson Ndoboli, Deo B. Ndumu, Eddie M. Wampande, Karyn A. Havas

**Affiliations:** ^1^Department of Public and Ecosystem Health, College of Veterinary Medicine, Cornell University, Ithaca, NY, United States; ^2^Center for Outcomes Research and Epidemiology, College of Veterinary Medicine, Kansas State University, Manhattan, KS, United States; ^3^Department of Political Science and Public Administration, College of Humanities and Social Sciences, Makerere University, Kampala, Uganda; ^4^Central Diagnostic Laboratory, College of Veterinary Medicine, Animal Resources and Biosecurity, Makerere University, Kampala, Uganda; ^5^Department of Animal Health, Ministry of Agriculture, Animal Industry and Fisheries, Entebbe, Uganda

**Keywords:** biosecurity practices, pig farmers, African swine fever, external biosecurity, internal biosecurity, questionnaire, pigs

## Abstract

**Introduction:**

In Uganda, pig production is an important source of livelihood for many people and contributes to food security. African swine fever (ASF) is a major constraint to pig production in Uganda, threatening the food supply and sustainable livelihoods. Prevention of ASF primarily relies on good biosecurity practices along the pig value chain. Previous studies showed that biosecurity along the pig value chain and on farms in Uganda is poor. However, the biosecurity practices of pig farmers in ASF affected areas of Uganda and their opinions on on-farm ASF morbidity and mortality were previously not comprehensively characterized. The objectives of this study were to document pig farmers’ experiences with ASF in their farms and to describe the pig biosecurity practices in districts of Uganda that were highly affected by ASF.

**Methods:**

A total of 99 farmers were interviewed in five districts. Data were collected by way of triangulation through farmer interviews, field observations during the farmer interviews, and a survey of key informants. However, farmer interviews were considered the primary source of data for this study. Farmers’ biosecurity practices were scored using a biosecurity scoring algorithm.

**Results:**

Forty-one out of 96 (42.7%) farmers reported having pigs with ASF in the past 12 months. The level of pig farming experience (*p* = 0.0083) and herd size (*p* < 0.0001) were significantly associated with the reported occurrence of ASF. Overall, the biosecurity scores for the respondents were considered poor with 99% (98/99) scoring <70% and just one farmer obtaining a fair score of 72.2%. District (*p* = 0.0481), type of husbandry system (*p* = 0.014), and type of pig breed raised (*p* = 0.004) were significantly associated with farmer’s biosecurity score.

**Conclusion:**

Continued farmer education on ASF and the importance of good biosecurity practices is necessary. More in-depth scientific inquiry into the factors influencing the biosecurity practices among pig farmers in Uganda is necessary.

## 1. Introduction

Pig production in Uganda is an important activity that contributes to national food security and provides a source of livelihood for many people (1). Implementation is largely informal and done by smallholder farmers with herd sizes typically ranging between 2 and 20 pigs (2). The national pig herd in Uganda was estimated to be 3.69 million in 2013 and 4.41 million pigs as of 2019 (3), highlighting an increase over the years due to the high demand for pork (1). The pig density is highest in central Uganda constituting 41.1% of the national pig herd, followed by the western region (24.4%), eastern region (22%) and northern Uganda (12.5%) (4). Despite the growth in the pig industry, African swine fever remains a major constraint to pig production in Uganda (5), where it is endemic (6), and is a threat to food security and sustainable livelihoods.

African swine fever (ASF) is a highly fatal viral disease of pigs whose prevention primarily relies on the strict implementation of biosecurity measures on pig farms (7, 8) and at all levels of the pig value chain (9). Biosecurity as applied to animal production has been defined as measures taken to prevent the introduction of disease into farms and to prevent the spread of disease within farms (10). Transmission of African swine fever virus (ASFV) in pig farming systems in sub-Saharan Africa, where it is endemic (11), occurs primarily due to lack of implementation of basic biosecurity measures. Despite the key role biosecurity measures play in disease prevention and control, implementation of these measures in many developing countries is a challenge due to factors related to lack of knowledge and awareness about biosecurity, financial constraints, and socio-cultural factors (12). Previous studies found that biosecurity along the pig value chain in Uganda is poor (5, 13, 14), and implementation of good biosecurity was constrained by the high financial cost of investment in biosecurity and the perception that such investment led to loss of income, lack of adequate land, and sociocultural barriers (15, 16). In addition to the biosecurity implementation barriers reported, a survey of smallholder pig farmers in northern Uganda showed that pig farmers’ willingness to invest in biosecurity reduced following ASF outbreaks and this resulted from their loss optimism about the preventive benefits of farm biosecurity (17).

Locally tailored solutions for ASF outbreaks that consider the multiplicity of factors influencing the uptake of good biosecurity practices may be needed for increased pig productivity. As an example, Dione and others recommended addressing the power disparities in family gender relations to ensure effective implementation of biosecurity (18). Additionally, an ethnographic study on biosecurity in the Mukono district of Uganda suggested that pig disease prevention measures should take into account the intimate role pigs play in the farmers’ livelihoods, because disease prevention in smallholder piggeries is not the only priority for farmers (19). Interventions targeting the prevention and control of ASFV require a comprehensive understanding of the pig production systems (5) and locally tailored solutions to ASF outbreaks require a good understanding of the existing pig husbandry practices. Although ASF is endemic in all regions of Uganda, our data from key pig slaughterhouses in peri-urban Kampala found most pigs with suspected ASF originated from districts primarily located in central Uganda. Farmers’ opinions on ASF morbidity and mortality and biosecurity practices of pig farmers in these selected districts of Uganda were previously not comprehensively characterized. The objectives of this study were to document pig farmers’ experiences with ASF in their farms and describe the biosecurity practices of pig farmers in selected districts of Uganda that were affected by ASF.

## 2. Materials and methods

### 2.1. Data sources, study sites, and participant selection

**Figure 1:**
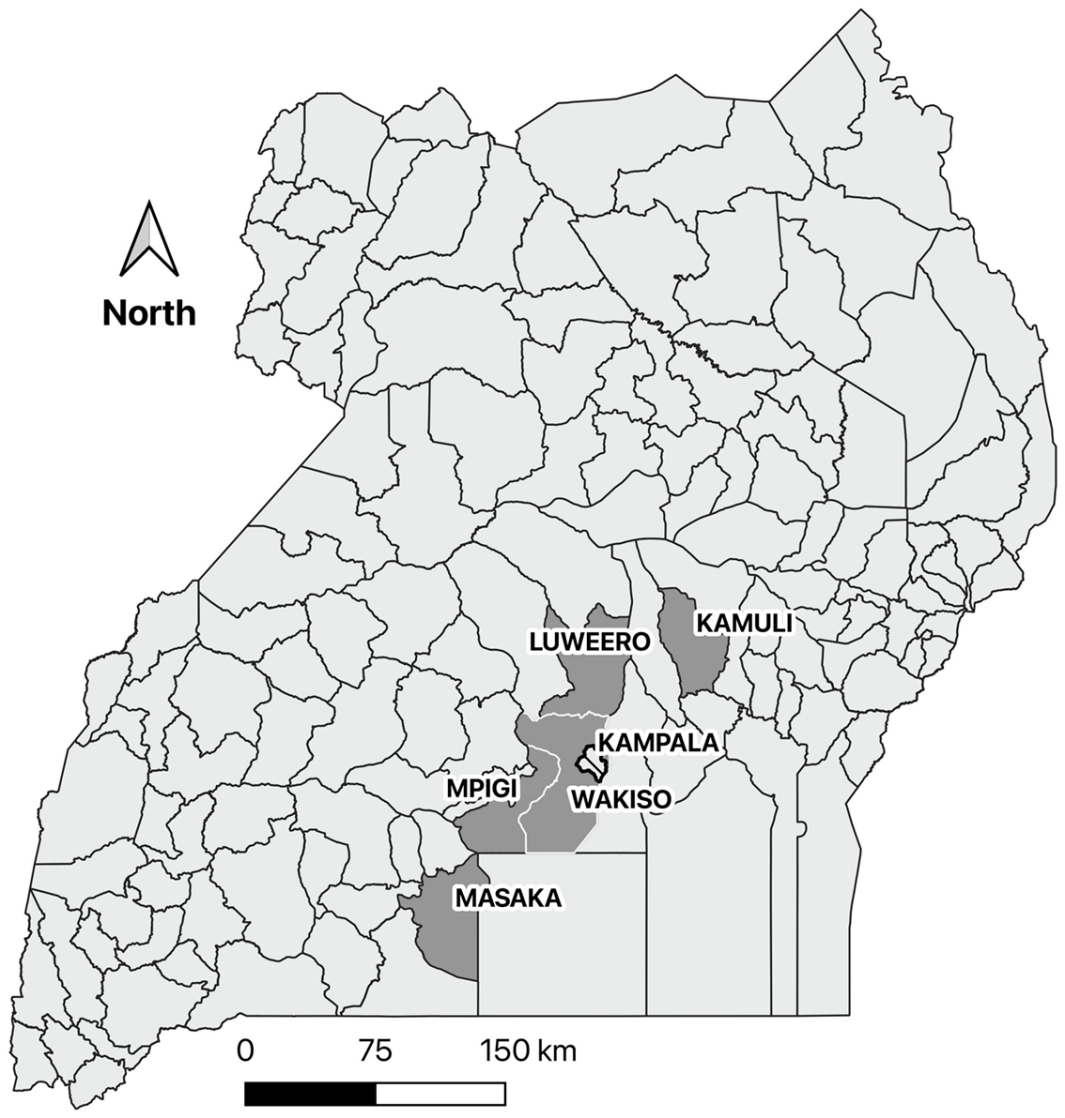
A map of Uganda showing the five selected districts most affected by African swine fever based on clinical and pathologic data collected from pigs slaughtered in the Kampala metropolitan area between May 2021 and June 2022. A survey of the biosecurity practices of pig farmers in these districts was conducted in June and July 2022.

Data were collected by way of triangulation through farmer interviews, field observations during the farmer interviews, and a survey of key informants. However, farmer interviews were considered the primary source of data for this study. The farmer interviews and field observations were conducted in four districts of central Uganda (Mpigi, Masaka, Luwero, and Wakiso) and one district in eastern Uganda (Kamuli) (Figure 1). These districts were selected based on data collected on clinical and pathologic lesions associated with ASF from pigs sampled from abattoirs around Kampala, Uganda. Districts with the highest number of pigs with splenic hemorrhages which is the most characteristic lesion of acute ASF (20) were selected. Convenience and purposive sampling were used to identify 19 to 20 respondents per district and sampling until saturation was used to identify trends (21, 22). The pig farmers who participated in the study were identified by the district veterinary officers (DVOs) and/or government animal health workers in each district with the intent of providing diversity in farmer gender, farm management styles, and farm size. The key informants were identified by the research team based at Makerere University and by the DVOs.

### 2.2. Questionnaire development and administration

Questionnaires were developed for the farmers and key informants, and a field observation checklist was developed as well. The farmer questionnaire and the key informant’s questionnaire were semi-structured and captured information on the farmer’s production practices, biosecurity practices, knowledge on ASF, previous experience with ASF, as well as impact of ASF on the farm, including mortality, and herd characteristics. Regarding previous ASF incursions and mortality, a simplified description of the clinical signs of ASF was provided to the respondent before the questions on ASF occurrence and the associated mortalities were asked. The questions on biosecurity were aligned with the good practices for pig biosecurity in developing and transition countries as described by the Food and Agriculture Organization (FAO) (23) and where applicable, as described by the University of Ghent Biocheck on-farm biosecurity assessment system (24).

The farmer questionnaire was first developed by the research team, and reviewed by two epidemiologists at Cornell University, and 11 experts on African swine fever in Uganda to ensure the questions were complete and response option adequate. Next, seven pig farmers in the central region, but not in districts enrolled in the study, pre-tested the farmer questionnaire and provided feedback on clarity. The questionnaire was modified based on the feedback from the pre-testing. The pre-tested farmer questionnaire was sent to the Makerere University Institute of Languages for translation from English into Luganda and Lusoga, which are the common native languages spoken in the study districts. The translated questionnaires were examined for validity of the translations and later back translated into English by a second individual who had not seen the original questionnaire to check for the accuracy in the translations. All the versions of the questionnaire (English, Luganda, and Lusoga) were built into KoBo Toolbox (Kobo Organization, Cambridge, MA, United States), and later downloaded into the KoBo collect app (Kobo Organization, Cambridge, MA, United States) for administration using computer tablets. The captured responses in the tablets were then uploaded to the KoBo online repository. The farmer questionnaire in English is provided as Supplementary File 1. The Luganda and Lusoga versions of this questionnaire will be made available upon request from the corresponding author.

The key informants’ questionnaire and the field observation checklist were built based on the farmer questionnaire to enable meaningful triangulation. The key informants’ questionnaire was also sent to a team of ASF experts in Uganda for pre-testing and modified to reflect the feedback from the pre-testing (Supplementary File 2). The observation checklist (Supplementary File 3) was modified after administration in the first district surveyed (Supplementary File 4). The key informants’ questionnaire was administered via Qualtrics (Qualtrics, Provo, UT, United States) for a period of 6 weeks (August 8 through September 22, 2022), and a reminder was sent to non-respondents every 2 weeks. The field observation checklist was also built into KoBo Toolbox and the data was collected using the KoBo Collect app in the data collection tablets.

The farmer interviews and field observations were conducted in the months of June and July 2022. The farmers were interviewed by two field research assistants from Makerere University in Uganda, and the field biosecurity observations were recorded by two field research assistants from Cornell University. All the field research assistants were trained prior to the onset of field data collection to ensure uniformity in the farmer interviews and field observations. Following the interviewer training, the farmer interviewers practiced administering the questionnaire. The interviews and the field observations were overseen by the primary author (JE). A biosecurity protocol was followed by the field data collection team during the farm visits. Each member of the field research team wore disposable boot covers between farms and applied hand sanitizers following each farm visit to ensure that the team did not disseminate pathogens between farms. The farmer interviews were conducted at the respondents’ homesteads/farms in a language that the respondent preferred. Responses captured in Luganda and Lusoga were later back translated into English. All the respondents consented to the interviews and field observation and each respondent was offered 20,000 Ugandan shillings (approximately USD 6 at the time) to reimburse for their time.

### 2.3. Biosecurity scoring scheme

For each biosecurity question, the risk was determined using a method previously described (25) and was as follows. First an overall value was assigned to the biosecurity measure the question asked about. Then, a weight was assigned to each of the response options that the farmer could choose for each question. A pig farmer who practiced good biosecurity scored all or almost all the points allocated to that specific question while one who did not, scored zero points. For binary response options, the farmer got all the points if the specific biosecurity aspect was practiced. However, responses measured on a Likert scale were weighted on a scale of 0 to 1, in increments of 0.25 as previously described (25). The farmer’s final scores for each question were obtained from the product of the selected answer weight and the biosecurity question value. For the Likert scale responses, a maximum score of one and a minimum score zero was deemed untenable for some biosecurity aspects depending on how the question was structured. For example, some questions had response options structured as “no, rarely, sometimes, and almost all of the time,” but with no “all of the time” option. Once all questions were scored, the sum of the scores was calculated and converted into a percentage. A biosecurity score of >90% was categorized as excellent, >80–90% as good, >70–80% as fair, and ≤ 70% as poor (25).

The values of different biosecurity measures were based on the Biocheck biosecurity scoring algorithm used at the University of Ghent and previously described (24). However, some biosecurity questions used in this study’s questionnaire either did not properly align or were not included in Ghent Biocheck scoring algorithm. As a result, the values provided by the Biocheck tool had to be validated for Ugandan pig farmers in the assessed districts. A total of 15 veterinarians evaluated the biosecurity value scheme and provided their expert opinion on the values or suggested alternative values for each biosecurity aspect in the questionnaire. Three members of the research team (JE, EM, and KH) were among the 15 veterinarians who evaluated the algorithm. The other 12 were practicing veterinarians in Uganda and included two veterinarians from each of the five districts surveyed and two academic veterinarians at Makerere University. The practicing veterinarians were selected based on their experience working with farmers in the study districts, and their knowledge of pig production and biosecurity in the selected districts. The 15 veterinarians were requested to either agree or disagree with the biosecurity measure value in the questionnaire as assigned by the Biocheck survey, and to assign a new value if they disagreed. They were also asked to assign a biosecurity measure value to aspects without any assigned values in the Biocheck tool. They provided an alternative value with a maximum score greater than zero and less than or equal to 10. The responses from all 15 veterinarians for each biosecurity aspect were summarized in Excel version 2303 Build 16.0.16227.20202 (Microsoft, Redmond, WA, United States) and the modal value for each biosecurity aspect was used in the final biosecurity scoring as it represented the most popular expert opinion. A summary of the modal values used to score farmer’s biosecurity is provided in Supplementary Table 1.

### 2.4. Data management and analysis

The data from the farmer interviews and field observations were downloaded from KoBo into Excel and collated. The key informant survey data was downloaded from Qualtrics into Excel as well. The data were analyzed using commercial statistical software (SAS, version 9.4, SAS Institute Inc., Cary, NC, United States). Data on common diseases affecting the farmers’ pigs was captured as free text and was analyzed qualitatively into two categories, farmers who mentioned ASF and/or at least one clinical sign of ASF, and those who did not. Categorical data were then summarized using frequencies and proportions, and confidence intervals for the proportions were calculated using the Agresti-Coull method (26). The Pearson chi-squared test was used to explore the association between district, years involved in pig farming, pig husbandry type, pig breed type, herd size, and source of farm labor with self-reporting of ASF in the past 12 months. Respondents who were not sure if their pigs had ASF in the past 12 months were excluded from the analysis. If the chi-squared test assumptions were violated, the Fisher’s exact test was used. Multiple comparisons of proportions following a significant chi-squared or Fisher’s exact test was conducted using a *post hoc* Tukey-style multiple comparisons test of proportions using the COMPPROP SAS macro (27).

Biosecurity scores were tested for normality using a visual assessment of a histogram and the Shapiro–Wilk test and they showed the data were normally distributed (*p* = 0.729). The mean, standard deviation, and range of the biosecurity scores were calculated. The one-way analysis of variance (one-way ANOVA) test was used to compare mean biosecurity scores between different groups of farmers by pig production type (farmers that use confinement in corrals versus all other husbandry systems), pig type (farmers who raised local breed pigs, mixed breed types, exotic breed pigs, and all other types), and between districts (Mpigi, Masaka, Luwero, Kamuli, and Wakiso). Levene’s test was used to check for the assumptions of homogeneity of variances. If the assumption of homogeneity of variances was not met, Welch’s ANOVA was used. Multiple pairwise comparisons were done if the ANOVA was significant and used a Tukey’s studentized range (HSD) test to control for type 1 experiment-wise error rate. The level of significance for all statistical tests was 0.05.

## 3. Results

### 3.1. Farmer respondents’ and farm characteristics

Overall, a total of 99 pig farmers were interviewed in the five districts and results are summarized in Table 1. Nineteen respondents were interviewed in Mpigi, and 20 in each of the districts of Masaka, Luwero, Kamuli and Wakiso. Overall, more males (56.6%, 56) were interviewed when compared to females (43.4%, 43), 39.8% (28) of farmers had spent five or less years raising pigs, and 63.6% (63) of farm labor was provided by family members.

**Table 1:** Characteristics of the farms included in the evaluation of biosecurity practices of pig farmers in selected districts of Uganda with high levels of suspect ASFV cases, June 2022 through July 2022.

Characteristics of farm/piggery	Mpigi*	Masaka	Luwero	Kamuli	Wakiso	Overall	*P*-value†
	Number (%) of respondents						
**Gender of respondents (*n* = 99)**	**19 (100)**	**20 (100)**	**20 (100)**	**20 (100)**	**20 (100)**	**99 (100)**	–
Female	12 (63.2)	10 (50)	11 (55)	4 (20)	6 (30)	43 (43.4)	
Male	7 (36.8)	10 (50)	9 (45)	16 (80)	14 (70)	56 (56.6)	
**Years in pig farming (*n* = 98)**	**19 (100)**	**20 (100)**	**20 (100)**	**20 (100)**	**19 (100)**	**98 (100)**	0.0083
≤5	9 (47.4)	7 (35)	10 (50)	6 (30)	7 (36.8)	39 (39.8)	
6–10	7 (36.8)	5 (25)	7 (35)	5 (25)	7 (36.8)	31 (31.6)	
11–20	1 (5.3)	7 (35)	3 (15)	4 (20)	5 (26.3)	20 (20.4)	
>20	2 (10.5)	1 (5)	0 (0)	5 (25)	0 (0)	8 (8.2)	
**Pig husbandry system (*n* = 99)**	**19 (100)**	**20 (100)**	**20 (100)**	**20 (100)**	**20 (100)**	**99 (100)**	0.564
Confinement in corrals	12 (63.2)	20 (100)	15 (75)	18 (90)	15 (75)	80 (80.8)	
Tethering	0 (0)	0 (0)	1 (5)	0 (0)	1 (5)	2 (2.0)	
Mixed, confined and tethered	4 (21)	0 (0)	4 (20)	2 (10)	3 (15)	13 (13.1)	
Mixed, confined and free-range	2 (10.5)	0 (0)	0 (0)	0 (0)	1 (5)	3 (3)	
Mixed, tethering and free-range	1 (5.3)	0 (0)	0 (0)	0 (0)	0 (0)	1 (1.0)	
**Herd size (*n* = 99)**	**19 (100)**	**20 (100)**	**20 (100)**	**20 (100)**	**20 (100)**	**99 (100)**	<0.0001
1–3 pigs	0 (0)	0 (0)	1 (5)	0 (0)	2 (10)	3 (3.0)	
4–11 pigs	8 (42.1)	4 (20)	12 (60)	9 (45)	6 (30)	39 (39.4)	
12–20 pigs	6 (31.6)	3 (15)	2 (10)	4 (20)	4 (20)	19 (19.2)	
21–30 pigs	3 (15.8)	5 (25)	3 (15)	1 (5)	4 (20)	16 (16.2)	
31–40 pigs	0 (0)	3 (15)	1 (5)	0 (0)	0 (0)	4 (4.0)	
41–50 pigs	0 (0)	0 (0)	0 (0)	2 (10)	1 (5)	3 (3.0)	
>50 pigs	2 (10.5)	5 (25)	1 (5)	4 (20)	3 (15)	15 (15.2)	
**Pig type raised (*n* = 99)**	**19 (100)**	**20 (100)**	**20 (100)**	**20 (100)**	**20 (100)**	**99 (100)**	0.804
Local	6 (31.6)	1 (5)	1 (5)	1 (5)	4 (20)	13 (13.1)	
Mixed	2 (10.5)	7 (35)	10 (50)	8 (40)	6 (30)	33 (33.3)	
Exotic/European	6 (31.6)	7 (35)	3 (15)	7 (35)	7 (35)	30 (30.3)	
Local & mixed	2 (10.5)	0 (0)	4 (20)	1 (5)	1 (5)	8 (8.1)	
Local & exotic	0 (0)	0 (0)	0 (0)	1 (5)	1 (5)	2 (2.0)	
Mixed and Exotic/European	2 (10.5)	5 (25)	1 (5)	1 (5)	1 (5)	10 (10.1)	
All the three groups (local, mixed, and exotic/European)	1 (5.3)	0 (0)	1 (5)	1 (5)	0 (0)	3 (3.0)	
**Source of farm labor (*n* = 99)**	**19 (100)**	**20 (100)**	**20 (100)**	**20 (100)**	**20 (100)**	**99 (100)**	0.321
Family members	13 (68.4)	9 (45)	18 (90)	12 (60)	11 (55)	63 (63.6)	
Both family and externally employed persons	4 (21.1)	5 (25)	1 (5)	5 (25)	6 (30)	21 (21.2)	
Externally employed persons	2 (10.5)	6 (30)	1 (5)	3 (15)	3 (15)	15 (15.2)	

* The husbandry system reported was based on researchers’ field observations.

†Results of the Chi-squared analysis of the associations of the variables with the reported occurrence of ASF sick pigs in the past 12 months.The bold values indicate the total number of respondents that replied to that question.

Among farm characteristics, 80.5% (80/99) of farmers across all districts confined their pigs in corrals. Mpigi had the greatest diversity in production systems with 63.2% (12/19) confining pigs, 21% (4/19) confining and tethering pigs, 10.5% (2/19) using confinement and free-range, and 5.3% (1/19) using tethering and free range. Farmers raised a variety of pig types as well. The three major pig types included exotic or European breed pigs, local Ugandan breed of pigs, and mixed breeds of the two. Farmers reported raising one type, two different types or all three types. In all districts, 33.3% (33/99) of the farmers raised mixed breed pigs and 30% (30/99) raised exotic pigs (Table 1). Mpigi farmers differed, the majority of them raised local breed pigs (31.9%; 6/19). There was diversity in the size of farm, but 39.4% (30.99) of farmers raised 4 to 11 pigs, followed by 19.2% (19/99) that raised 12 to 20, 16.2% (16/99) that raised 21 to 30, and 15.2% (15/99) that raised >50. Less than 5% raised 1 to 3, 31 to 40, and 41 to 50 pigs. Masaka and Mpigi had more farmers raising >50 pigs, 25% (5/20) and 20% (4/20), respectively.

### 3.2. Farmers’ opinions on common diseases, and ASF morbidity and mortality

When asked about the common diseases affecting their pigs, 82.8% of the 99 farmers interviewed mentioned ASF and/or at least one clinical sign of ASF, 12.1% (12) did not mention ASF and/or at least one clinical sign of ASF, 4% (4) reported they had not experienced disease on their farms, and 1% (1) did not know common diseases on their farm. There were 84 (84.9%) farmers that stated they had knowledge about a disease of pigs called ASF. When asked about ASF occurrence, 42.7% (41/96) reported having ASF sick pigs in the past 12 months, and 3% (3/99) were unsure if their pigs had ASF in the past 12 months.

There were 41 farmers who reported having pigs with ASF. Among them, 85.4% (29) said that some or all the pigs with ASF died. Four out of 19 (21.1%) farmers in Mpigi reported having pigs with ASF in the past 12 months, 45% (9/20) in Masaka, 50% (10/20) in Luwero, 30% (6/20) in Kamuli, and 60% (12/20) in Wakiso. Significant omnibus associations were found between reporting ASF on farms in the past 12 months and farming experience (*p* = 0.0083) as well as herd size (*p* < 0.0001), but no post-hoc pairwise significant differences were found.

#### 3.2.1. External biosecurity

Farmers were asked about a variety of external biosecurity components including wild animal threats and fencing. No farmer from any of the districts reported seeing wild pigs such as warthogs or bush pigs in the past 12 months within or near their farms. No farmer reported contact of their pigs with wild pigs in the past 12 months. Overall, 37.5% (37/99) of the farmers said they had a fence around their pigs as a physical biosecurity barrier. Kamuli district had the most farmers (75%; 15/20) that reported the presence of fence, followed by Masaka (35%; 7/20), Wakiso (35%; 7/20), Mpigi (31.6%; 6/19), and then Luwero (10%; 2/20). A detailed breakdown of the farmers responses on physical biosecurity barriers and herd status is provided in Table 2.

**Table 2:** Pig farmers’ responses regarding the presence of a physical biosecurity barrier, introduction, and segregation of arriving pigs in selected districts of Uganda with high levels of suspect ASFV cases, June 2022 through July 2022.

	Number (%) of respondents					
	Mpigi	Masaka	Luwero	Kamuli	Wakiso	All districts
**Biosecurity category: physical biosecurity barriers**						
Have a fenceɎ	6 (31.6)	7 (35)	2 (10)	15 (75)	7 (35)	37 (37.4)
**Introduction of animals to the farm/piggery**						
Some or all replacement female pigs obtained from outside farms/piggeries‡	10 (55.6)	15 (75)	13 (65)	11 (57.9)	10 (50)	59 (60.8)
Some or all boars for mating are obtained from outside farms/piggeries†	13 (72.2)	12 (75)	17 (89.5)	12 (66.7)	12 (63.2)	66 (73.3)
Some or all weaned pigs obtained from outside farms/piggeries¥	2 (10.5)	6 (46.2)	6 (42.9)	8 (57.1)	8 (53.3)	30 (40)
Replacement females examined for health by a veterinary professional before introduction*	5 (27.8)	11 (68.8)	10 (58.8)	5 (35.7)	5 (41.7)	36 (46.8)
Weaned pigs examined for health by a veterinary professional before introduction**	8 (42.1)	7 (53.9)	3 (25)	3 (23.1)	2 (18.2)	23 (33.8)
Boars for mating are examined for health by a veterinary professional prior to introduction***	4 (30.7)	4 (33.3)	6 (35.3)	4 (33.3)	7 (58.3)	25 (37.9)
**Biosecurity category: segregation of arriving pigs**						
**Newly introduced pigs quarantined**	**19 (100)**	**20 (100)**	**20 (100)**	**20 (100)**	**20 (100)**	**99 (100)**
No	13 (68.4)	5 (25)	11 (55)	9 (45)	10 (50)	48 (48.5)
Rarely	0 (0)	3 (15)	0 (0)	1 (5)	1 (5)	5 (5.1)
Sometimes	3 (15.8)	2 (10)	2 (10)	0 (0)	3 (15)	10 (10.1)
Almost all the time	2 (10.5)	10 (50)	7 (35)	10 (50)	6 (30)	35 (35.4)
Not sure	1 (5.3)	0 (0)	0 (0)	0 (0)	0 (0)	1 (1)
**Animals returned from the market are kept with other pigs**	**19 (100)**	**20 (100)**	**19 (100)**	**19 (100)**	**20 (100)**	**97 (100)**
No	18 (94.7)	20 (100)	19 (100)	19 (100)	20 (100)	96 (99)
Rarely	0 (0)	0 (0)	0 (0)	0 (0)	0 (0)	0 (0)
Sometimes	0 (0)	0 (0)	0 (0)	0 (0)	0 (0)	0 (0)
Almost all the time	1 (5.3)	0 (0)	0 (0)	0 (0)	0 (0)	1 (1)

ɎNumber of respondents: Mpigi = 19, Masaka = 20, Luwero = 20, Kamuli = 20, Wakiso = 20, all districts = 99.

‡Number of respondents: Mpigi = 18, Masaka = 20, Luwero = 20, Kamuli = 19, Wakiso = 20, all districts = 97.

†Number of respondents: Mpigi = 18, Masaka = 16, Luwero = 19, Kamuli = 18, Wakiso = 19, all districts = 90.

¥Number of respondents: Mpigi = 19, Masaka = 13, Luwero = 14, Kamuli = 14, Wakiso = 15, all districts = 75.

* Number of respondents: Mpigi = 18, Masaka = 16, Luwero = 17, Kamuli = 14, Wakiso = 12, all districts = 77.

** Number of respondents: Mpigi = 19, Masaka = 13, Luwero = 12, Kamuli = 13, Wakiso = 11, all districts = 68.

*** Number of respondents: Mpigi = 13, Masaka = 12, Luwero = 17, Kamuli = 12, Wakiso = 12, All districts = 66.The bolded values indicate the total number of respondents that replied to that question.

Farmers were also asked about how they kept unhealthy animals off their farm. In reference to replacement females and their boars, more than 50% of the farms had open herds and, for weaned pigs, 40% (30/75) of farmers obtained them from sources outside the farm. Among all farms, 46.8% (36/77) of those that brought replacement females from outside sources had them examined by a veterinary professional prior to bringing them onto a farm. There were 33.8% (23/68) and 37.9% (25/66) of farmers that reported pre-acquisition health assessments by veterinary professionals for weaned pigs and boars, respectively. Overall, 48.5% (48/99) of the farmers reported they did not quarantine newly acquired pigs and 35.4% (35/99) quarantined newly introduced pigs almost all the time. The other 15.1% either quarantined rarely or sometimes and 1% (1) was not sure of the farms quarantine practices. Only one out of 97 respondents reported that animals returned from the market were mixed with the rest of the herd. A detailed summary of the responses regarding segregation of arriving pigs for each district surveyed is provided in the Table 2.

Farmers were also asked how they managed indirect transmission threats from other farms through shared equipment and their own contact with other pigs. Only 2% (2/99) of the respondents said they shared their farm equipment with other farmers and the shared equipment was not cleaned and disinfected between farms. Twenty-eight out of 99 (28.3%) respondents reported that persons in contact with their pigs did not have contact with other farmers’ pigs. However, 10.1% (10/99) had contact with other farms’ pigs almost all the time, 41.4% (41/99) sometimes had contact with other farms’ pigs, and 15.2% (15/99) rarely did so. A breakdown of the respondents’ answers regarding farm personnel and farm equipment sharing across the surveyed districts is provided in Table 3.

**Table 3:** A summary of the respondents’ answers regarding farm equipment sharing, farm personnel, presence of footbaths, and farm visitors across the surveyed districts of Uganda with high levels of suspect ASFV cases, June 2022 through July 2022.

	Number (%) of respondents					
	Mpigi	Masaka	Luwero	Kamuli	Wakiso	All districts
**Biosecurity category: farm equipment sharing**						
Farm equipment shared with other farmers (*n* = 99)	1 (5.2)	0 (0)	0 (0)	0 (0)	1 (5)	2 (2)
**Biosecurity category: farm personnel & footbaths**						
**Persons in contact with farm’s pigs have contact with other pigs**	**19 (100)**	**20 (100)**	**20 (100)**	**20 (100)**	**20 (100)**	**99 (100)**
No	7 (36.8)	4 (20)	0 (0)	10 (50)	7 (35)	28 (28.3)
Rarely	3 (15.8)	6 (30)	2 (10)	3 (15)	1 (5)	15 (15.2)
Sometimes	6 (31.6)	8 (40)	13 (65)	4 (20)	10 (50)	41 (41.4)
Almost all the time	2 (10.5)	2 (10)	2 (10)	3 (15)	1 (5)	10 (10.1)
Not sure	1 (5.3)	0 (0)	3 (15)	0 (0)	1 (5)	5 (5)
**Footbaths and disinfectants present**	**17 (100)**	**15 (100)**	**17 (100)**	**16 (100)**	**17 (100)**	**82 (100)**
No	13 (76.5)	8 (53.3)	16 (94.1)	11 (68.7)	12 (70.6)	60 (73.2)
Rarely	1 (5.9)	0 (0)	1 (5.9)	2 (12.5)	0 (0)	4 (4.9)
Sometimes	3 (17.7)	5 (33.3)	0 (0)	3 (18.8)	2 (11.8)	13 (15.9)
Almost all the time	0 (0)	2 (13.2)	0 (0)	0 (0)	3 (17.6)	5 (6)
**Biosecurity category: visitors**						
Category of farm visitors*	**16 (100)**	**15 (100)**	**17 (100)**	**16 (100)**	**17 (100)**	**81 (100)**
Animal health workers	16 (100)	15 (100)	16 (94.1)	15 (93.8)	17 (100)	79 (97.5)
Pig buyers	12 (75)	14 (93.3)	14 (82.4)	10 (62.5)	12 (70.6)	62 (76.5)
Other family members from outside the farm	8 (50)	8 (53.3)	10 (58.8)	4 (25)	5 (29.4)	35 (43.2)
Neighbors	8 (50)	3 (20)	5 (29.4)	1 (6.5)	5 (29.4)	22 (27.2)
Community leaders	1 (6.3)	2 (13.3)	3 (17.7)	5 (31.3)	2 (11.8)	13 (16)
**Visitors go to the area pigs are kept**	**19 (100)**	**20 (100)**	**20 (100)**	**20 (100)**	**20 (100)**	**99 (100)**
No	3 (15.8)	5 (25)	3 (15)	4 (20)	3 (15)	18 (18.2)
Rarely	5 (26.3)	3 (15)	4 (20)	3 (15)	5 (25)	20 (20.2)
Sometimes	7 (36.8)	11 (55)	11 (55)	10 (50)	11 (55)	50 (50.5)
Almost all the time	4 (21.1)	1 (5)	2 (10)	3 (15)	1 (5)	11 (11.1)
**Visitors use farm specific clothes and footwear**	**18 (100)**	**15 (100)**	**17 (100)**	**16 (100)**	**17 (100)**	**83 (100)**
No	18 (100)	13 (86.6)	16 (94.1)	14 (87.5)	17 (100)	78 (94)
Rarely	0 (0)	0 (0)	0 (0)	0 (0)	0 (0)	0 (0)
Sometimes	0 (0)	1 (6.7)	1 (5.9)	2 (12.5)	0 (0)	4 (4.8)
Almost all the time	0 (0)	1 (6.7)	0 (0)	0 (0)	0 (0)	1 (1.2)
**Visitors clean their footwear before contact with pigs**	**16 (100)**	**13 (100)**	**16 (100)**	**14 (100)**	**17 (100)**	**76 (100)**
No	11 (68.8)	9 (69.2)	14 (87.5)	12 (85.7)	15 (88.2)	61 (80.2)
Rarely	3 (18.8)	1 (7.7)	0 (0)	0 (0)	0 (0)	4 (5.3)
Sometimes	2 (12.5)	2 (15.4)	2 (12.5)	2 (14.3)	0 (0)	8 (10.5)
Almost all the time	0 (0)	1 (7.7)	0 (0)	0 (0)	2 (11.8)	3 (4)

* This were responses from a multiple answer question. The respondent could select more than one option. Therefore, the percentage for each category of visitor is out of the total number of respondents for this question. The bolded values indicate the total number of respondents that replied to that question.

Farmers were also asked about how visitors were managed and what disease control tools were used. Of the 99 farms visited, 11.1% (11) allowed visitors to go to the area where pigs are kept almost all the time, 50.5% (50) sometimes, 20.2% (20) rarely, and only 18.2% (18) did not allow visitors in pig keeping areas. Seventy-eight out of 83 (94%) did not provide visitors with farm-specific clothing and footwear. For farms that did not provide farm-specific clothing and footwear, visitors did not clean their footwear before contact with pigs in 80.2% (61/76) farms. Disinfection footbaths were not present in 73.2% (60/82) farms either. When asked about the categories of visitors that contacted the farm’s pigs, 16% (13/81) were community leaders, 27.2% (22/81) were neighbors, 43.2% (35/81) were other family members from outside the farm, 76.5% (62/81) were pig buyers, and 97.5% (79/81) were animal health workers. A detailed description of the farmer responses regarding on-farm visitors, and fomite transmission management is provided in Table 3.

On most farms, farmers’ pigs did not mix with other pigs in the neighborhood (88.9%; 88/99), only 11.1% (11/99) reported that their pigs rarely or sometimes mixed with other pigs in the neighborhood. Most farmers reported that dogs did not have contact with the farm’s pigs (46.5%; 46/99), but 15.1% (15/99) of farmers reported that dogs contacted the pigs almost all the time, 26.3% (26/99) sometimes, and 9.1% (9/99) on rare occasions. Cats were reported to have direct contact with pigs almost all the time in 13.1% (13/99) of the farms, sometimes in 16.2% (16/99), and rarely in 10.1% (10) of the farms visited. The majority of the respondents (71.7%; 71/99) reported that other livestock did not have direct contact with farm’s pigs. About half of the respondents (51.5%; 51/99) reported that poultry had direct contact with their pigs almost all the time, 23.2% (23/99) sometimes, 6.1% (6/99) rarely, and 19.2% (19/99) said poultry did not have direct contact with their pigs. Twenty-two out of 99 farmers controlled flies around their pigs almost all the time, 44.4% (30) sometimes, 6.1% (6) rarely controlled flies, and 27.3% (27) did not control flies. Most farmers (61%; 60/99) did not control rodents around pigs. The distribution of the responses on contact of farm pigs with other domestic animals and poultry, and rodent and fly control across the five surveyed districts is provided in Supplementary Table 2.

Feeding of pigs on household leftovers was reported across the five districts with 56.6% (56/99) of the responses reporting this practice. Meat scraps were sometimes present in the household leftovers fed to pigs on 41% (23/56) of farms, rarely in 10.7% (6/56), almost all the time in just 3.6% (2), and 42.9% (24/56) of farms reported no meat scraps. The majority of farmers (82.1%; 46/56) that fed their pigs on household leftovers did not boil or re-cook the leftovers before feeding. Feeding pigs on restaurant leftovers was reported by 19.2% (19/99) of the respondents, but no farmers from Kamuli district reported this practice. Of the 19 that fed restaurant leftovers, 36.8% (7) and 47.4% (9) reported that meat scraps were present sometimes and almost all the time, respectively. Most farmers (73.6%; 14/19) reported that the restaurant leftovers were not boiled or re-cooked before feeding the pigs. Table 4 gives a detailed description of distribution of responses regarding swill feeding across the five districts.

**Table 4:** Distribution of pig farmers responses regarding swill feeding in selected districts of Uganda with high levels of suspect ASFV cases, June 2022 through July 2022.

Biosecurity category: swill feeding	Number (%) of respondents					
	Mpigi	Masaka	Luwero	Kamuli	Wakiso	All districts
Total number of respondents per district	19 (100)	20 (100)	20 (100)	20 (100)	20 (100)	99 (100)
**Fed pigs on household leftovers**	**15 (79)**	**8 (40)**	**15 (75)**	**7 (35)**	**11 (55)**	**56 (56.6)**
Meat scraps present in the household leftovers	15 (100)	8 (100)	15 (100)	7 (100)	11 (100)	56 (100)
No	4 (26.6)	3 (37.5)	9 (60)	5 (71.4)	3 (27.3)	24 (42.9)
Rarely	4 (26.6)	0 (0)	2 (13.3)	0 (0)	0 (0)	6 (10.7)
Sometimes	4 (26.6)	5 (62.5)	4 (26.7)	2 (28.6)	8 (72.7)	23 (41)
Almost all the time	2 (13.3)	0 (0)	0 (0)	0 (0)	0 (0)	2 (3.6)
Not sure	1 (6.7)	0 (0)	0 (0)	0 (0)	0 (0)	1 (1.8)
Household leftovers boiled or cooked again before feeding	15 (100)	8 (100)	15 (100)	7 (100)	11 (100)	56 (100)
No	13 (86.6)	4 (50)	13 (86.6)	5 (71.4)	11 (100)	46 (82.1)
Rarely	0 (0)	1 (12.5)	0 (0)	0 (0)	0 (0)	1 (1.8)
Sometimes	1 (6.7)	0 (0)	1 (6.7)	1 (14.3)	0 (0)	3 (5.4)
Almost all the time	1 (6.7)	3 (37.5)	1 (6.7)	1 (14.3)	0 (0)	6 (10.7)
**Fed pigs on restaurant leftovers**	**6 (31.6)**	**4 (20)**	**5 (25)**	**0 (0)**	**4 (20)**	**19 (19.2)**
Meat scraps present in the restaurant leftovers	6 (100)	4 (100)	5 (100)	0 (0)	4 (100)	19 (100)
No	0 (0)	0 (0)	3 (60)	0 (0)	0 (0)	3 (15.8)
Rarely	0 (0)	0 (0)	0 (0)	0 (0)	0 (0)	0 (0)
Sometimes	2 (33.3)	1 (25)	2 (40)	0 (0)	2 (50)	7 (36.8)
Almost all the time	4 (66.7)	3 (75)	0 (0)	0 (0)	2 (50)	9 (47.4)
Restaurant leftovers boiled or cooked again before feeding	6 (100)	4 (100)	5 (100)	0 (0)	4 (0)	19 (100)
No	5 (83.3)	1 (25)	4 (80)	0 (0)	4 (100)	14 (73.6)
Rarely	0 (0)	1 (25)	0 (0)	0 (0)	0 (0)	1 (5.3)
Sometimes	1 (16.8)	0 (0)	0 (0)	0 (0)	0 (0)	1 (5.3)
Almost all the time	0 (0)	2 (50)	1 (20)	0 (0)	0 (0)	3 (15.8)

The bold values indicate the total number of respondents that replied to that question.

#### 3.2.2. Internal biosecurity

Cleaning and sanitation within a farm aids disease control and questions were asked about on-farm practices (Supplementary Table 3). Overall, a large proportion of respondents (44.4%; 44/99) did not wash their hands before working with pigs, 4% (4/99) rarely did, 22.2% (22/99) sometimes did, but 29.3% (29/99) of farmers washed their hands almost all the time before working with their pigs. Most farmers (65%; 64/99) did not use specific clothing when working with their pigs. Yet, using specific footwear had almost evenly divided responses. There were 41.4% (41/99) of farmers that did not use footwear dedicated to working with their pigs, but there was 40.4% (40/99) of farmers that did. All the respondents (100%; 99/99) stated they regularly cleaned their pig pens and pig holding areas. Only 6.1% (6/99) reported using a disinfectant during cleaning and 33.3% (2/6) specifically reported using bleach (sodium hypochlorite).

For all the districts surveyed, respondents reported multiple ways they managed sick pigs on their farms (Supplementary Table 4). The primary response among all farmers in all districts was that sick pigs were treated (90%; 89/99), the next most common response was that they were isolated (64.6%; 64/99), then sold (21.2%; 21/99) and a few farmers in Luwero (1/20), Kamuli (1/20), and Wakiso (2/20) reported selling and consuming pork from sick pigs (4%; 4/99). Many farmers took a combination of actions when dealing with sick pigs, with the most common combination being the treatment and isolation of pigs.

Questions about mortality management were also asked (Supplementary Table 4). More than half of the respondents (56%; 51/91) buried dead pigs almost all the time, 13.2% (12/91) sometimes buried, 5.5% (5/91) rarely buried, and 25.3% (23/99) did not bury them. Most farmers (93.4%; 85/91) did not burn dead pigs, feed pigs to dogs (90.1%; 82/91), or throw them out into the surrounding environment (86.8%; 79/91). Although there were a handful of individuals that did all these things at least occasionally. Only one individual out of 91 (1.1%) reported that they rarely sold pork from dead pigs, and another (1.1%) reported that they sometimes sold pork from dead pigs.

### 3.3. Biosecurity practices based on field observations

The summary of field observations had to be different for Mpigi compared to all the other districts, because it was necessary to modify the observational checklist while in Mpigi, the first district to be surveyed. The observations were able to support that on the 19 farms visited in Mpigi, it was observed that human food leftovers were in feeding troughs in 31.6% (6) of the farms and 50% (3/6) had meat scraps. Livestock (57.9%; 11), dogs and cats (26.3%; 5), as well as flies and rodents (89.5%; 17) had some contact with the pigs, as did some visitors that came by (15.8%; 3). It was observed that the majority of pig pens appeared clean (89.5%; 17). It appeared that 73.7% (14) spread pig manure to nearby crop fields or gardens, and no farms had a hand washing facility near the area where pigs were kept. No wild pigs or *Ornithodoros* species of tick were seen either. A detailed breakdown of the biosecurity aspects observed by the research team in Mpigi District is provided in Supplementary Table 5.

For the rest of the four districts (Masaka, Luwero, Kamuli, and Wakiso), human food leftovers were observed in 11.4% (9/79) of the farms visited, but none were observed in Kamuli or Wakiso. Only one farm in Luwero (11.1%) of the nine farms with food leftovers had meat scraps. There was a fence on 23.8% of farms (19) and 89.5% (17/19) surrounded the entire property. Hand washing (31.6%; 6/19) and farm-specific clothing (5.3%; 1/19) were most observed in Masaka, and no farm-specific footwear was seen in any district. All districts had some observations of cats and dogs mixing with pigs (61.5%; 49/80); poultry mingling with pigs (78.8%; 63/80), and other livestock mingling with pigs (35%; 28/80). Thirty-six out of 80 (45%) farms visited spread pig manure to nearby crop fields or gardens, and this was observed on some farms from every district. Thirty-three out of 79 farms (41.8%) had an enclosure or area that could be dedicated for quarantine of new pigs. There were some visitors seen to have contact with pigs as well (3.8%; 3/80), but it was not commonly observed (80.5%; 70/80). A detailed description of the field observations in the districts of Masaka, Luwero, Mpigi, and Wakiso is provided in Supplementary Tables 6A,B.

### 3.4. Key informants’ opinions regarding pig farmers’ biosecurity practices

Of the 54 invited key informants, 35.2% (19/54) took the survey and completed some or all the questions in the questionnaire. In total, 84.2% (16/19) were local government employees, one was an employee of the agriculture ministry, one worked in academia, and the other worked in the private sector. Of the 16 local government key informants, 81.3% (13) worked in Wakiso district, 12.5% (2) were from Masaka, and 6.2% (1) was from Luwero. There were no key informant respondents from the districts of Mpigi or Kamuli (Supplementary Table 7).

Key informants reported on similar biosecurity methods asked about in the farmer questionnaires. As for external biosecurity, 63.2% (12/19) of key informants reported that only 1 to 25% of the farmers they interacted with quarantined new animals before their introduction to other pigs on the farm. Only 11.1% (2/18) of key informants reported that it was very common for farmers to involve animal health workers in evaluating the pre-acquisition health status of pigs, 44.4% (8/18) reported that this practice was both common and not common. There was 68.4% (13/19) of key informants that reported that use of household waste as feed was very common among pig farmers and 33.3% (6/18) mentioned that it was very common for farmers to feed their pigs on restaurant waste. The most common response among key informants was that 1–25% of farmers boiled swill before feeding it to their pigs (38.9%;7/18), and the next most common was that no farmers boiled swill before feeding it to pigs (27.8%; 5/18). Most key informants also reported that no farmers or 1–25% of farmers used or had visitors wear farm specific clothing or shoes. The most commonly reported visitors were the animal health worker (72.2%; 13/18) and the pig buyer (50%; 9/18). Among key informants, 50% (9/18) said it was common for farmers to isolate and treat sick pigs, 70.6% (12/18) said it was common for farmers to consume pork from sick pigs, and 50% (9/18) said it was common for farmers to sell pork from sick pigs. Most key informants also stated it was very common for farmers to sell off sick pigs (50%; 9/18). The majority of the key informants (77.8%; 14/18) mentioned that just 1–25% of pig farmers provided footbaths with disinfectant at the entrance to pig housing. Supplementary Tables 8A,B provide a detailed description of the key informant’s answers regarding pig farmers’ biosecurity practices.

### 3.5. Biosecurity scores

Table 5 provides a summary of the biosecurity mean scores of farmers. Overall, the biosecurity scores for the respondents were considered poor with 98/99 (99%) scoring <70%. The mean biosecurity score for all the respondents was 49% (standard deviation (SD) = 8.5%, range: 29.3–72.2%). There was a statistically significant difference in biosecurity scores between districts (*p* = 0.0481). The mean biosecurity score for Kamuli district of 52.6% (SD = 6.5%, range: 41.9–63.8%) was significantly higher than for Luwero (44.9%; SD = 6.2%, range: 33.6–57.3%) with a difference in means of 7.72% (95% confidence interval (95%CI): 0.46–14.99%).

**Table 5:** Distribution of pig farmers biosecurity scores in selected districts of Uganda with high levels of suspect ASFV cases, June 2022 through July 2022.

			Biosecurity scores (out of 100)			*P*-value^ǂ^
	Number of respondents	Mean	Standard deviation	Minimum	Maximum	
**Overall (all respondents)** ^¥^	99	49	8.5	29.3	72.2	
**District**						0.0481
Mpigi	19	49.9	9	33.2	63.8	
Masaka	20	50.4	7	38.3	64.2	
Luwero^¥^	20	44.9	6.2	33.6	57.3	
Kamuli^¥^	20	52.6	6.5	41.9	63.8	
Wakiso	20	47.8	11.4	29.3	72.2	
**Years in pig farming**						0.921
≤5	39	49	8.1	33.6	64.2	
6–10	31	49.7	9.8	29.7	72.2	
11–20	20	48.6	7.2	29.3	59.7	
>20	8	50.7	8.5	38.7	63.8	
**Pig husbandry system**						0.014
Confinement in corrals (intensive)	80	50.1	8.5	29.3	72.2	
All other types	19	44.8	7.2	29.7	60.1	
**Herd size**						
1–3 pigs	3	38	3.7	35.8	42.3	
4–11 pigs	39	47	7.3	29.7	61.1	
12–20 pigs	19	50.9	8.7	29.3	63.8	
21–30 pigs	16	46.5	7.5	33.5	62.2	
31–40 pigs	4	45.9	6.2	39.4	54.1	
41–50 pigs	3	51.4	10.7	45.1	63.8	
>50 pigs	15	57.9	6.4	44.5	72.2	
**Pig type raised**						0.004
Local	13	43.6	7.2	29.7	60.1	
Mixed	33	50.5	7.5	37.9	64.2	
Exotic/European	30	52.1	8.6	37.4	72.2	
All other types	23	46.3	8.6	29.3	61.5	
**Source of farm labor**						0.097
Family members	63	47.8	8.2	29.7	63.8	
Both family and externally employed persons	21	50.4	7.6	29.3	63.8	
Externally employed persons	15	52.8	10.2	33.6	72.2	

^¥^98/99 (99%) had a biosecurity score < 70%.

^ǂ^Results from the analysis of variance test.

^¥^Only the mean scores for these two districts were significantly different.

The statistical differences found include the following. The mean scores among farmers that used confinement compared to other husbandry systems were significantly different (*p* = 0.014). Confinement had a score of 50.1% (SD = 8.5%: 29.3–72.2%) and all other husbandry systems had a score of 44.8 (SD = 7.2%, range: 29.7–60.1%) with a difference in means of 5.3% (95%CI: 1.1–9.5%). For pig breed type, there was a statistical difference in scores between farmers that raised only exotic breed and those that raised only local breeds (*p* = 0.004). Farmers who raised only exotic pigs had a significantly higher mean score (52.1%; SD = 8.6%, range: 37.4–72.2%) when compared to those who raised local breed pigs (43.6%; SD = 7.2% range: 29.7–60.1%) with a difference in means of 8.5% (95%CI: 1.5–15.5%). Similarly, farmers who raised mixed breed pigs had significantly higher biosecurity mean score (50.5%; SD = 7.5%, range: 37.9–64.2%) when compared to those who raised local pigs giving a difference in means of 7% (95%CI: 0.06–13.9%).

## 4. Discussion

In the present study, pig biosecurity across the five districts was poor with just one farmer getting a fair biosecurity score. This key finding is similar to the findings of previous studies conducted in different parts of Uganda that reported poor pig biosecurity (5, 13, 14, 31). Comparing our findings to those in other regions of Uganda, a study that evaluated farm biosecurity in a medium-sized farm in northern Uganda found that biosecurity was largely lacking (31). This is similar to studies conducted in other countries of East Africa. A study conducted in Tanzania found poor biosecurity practices across pig raising communities in the southern highland of that country (32). Outside of Africa, poor biosecurity scores were found in ASF outbreak farms in Estonia (33), and a previous study found minimal application of pig biosecurity in eastern Indonesia (34). However, in Spanish herds, medium sized and large farms had higher biosecurity when compared to small herds in low pig density areas (35). In general, the risk of disease introduction and spread is higher in farms with poor biosecurity (36). The poor biosecurity found in the present study could be associated with the nature of the pig production system in Uganda which is largely a low-input/low-output operation (12) and could perhaps reflect on farmers’ unwillingness to invest in biosecurity because of the associated costs and lack of appreciation of the immediate benefits of good biosecurity. An ex-ante study that evaluated the impacts of biosecurity interventions on the control of ASF outbreaks In Masaka district of Uganda reported that although biosecurity interventions could reduce ASF outbreaks, their implementation could also lead to loss of income among farmers as an unintended consequence (37).

Based on our findings, farmer educational programs by veterinary extension officials on good biosecurity practices may be necessary. Participatory approaches to improving biosecurity through establishment of community biosecurity contracts that have been piloted in northern Uganda (29) could be trialed in the districts surveyed in the present study. However, acquisition of biosecurity knowledge alone may not guarantee farmers’ adoption and implementation of good biosecurity practices. A study conducted in Masaka and Lira districts of Uganda showed that the biosecurity knowledge of pig farmers improved but the biosecurity practices did not change following the training. The main reasons given for the failure in implementation of good biosecurity were the high financial costs, lack of adequate land, and sociocultural barriers (15). Infrastructural constraints, high cost of investment in biosecurity and the associated loss of income, social norms and traditions were barriers to good biosecurity in northern Uganda as well (16). It has been shown that a complex set of factors such as the farmers’ individual characteristics, costs associated with establishing and implementing effective biosecurity (38), disparities in gender roles between men and women (18), and psychosocial factors influence farmers’ adoption and adherence to good biosecurity practices (39). More in-depth scientific inquiry into the factors influencing the biosecurity practices of pig farmers in Uganda is necessary and should include an exploration of the possible psychosocial drivers.

Despite the poor biosecurity scores reported in the present study, we found significant difference in the biosecurity scores of two districts, among pig husbandry systems, and pig breed types raised. The mean biosecurity score for Kamuli district was significantly higher than for Luwero despite overall poor biosecurity scores in these districts. Perhaps, this observed difference could be due to a higher level of investment by Kamuli farmers on biosecurity in response to past ASF outbreaks and experiences. This difference might also reflect differences in biosecurity knowledge among the farmers in these districts resulting from more farmer education on biosecurity by district veterinary extension staff and/or by non-governmental organizations operating in Kamuli. Additionally, the influence of sociocultural barriers on the implementation of biosecurity measures might vary by district. Our findings show that the mean biosecurity score for farms that solely confined pigs in corrals was higher than that for all other husbandry systems. This finding is similar to the findings of a study conducted in Cameroon that found significantly lower biosecurity scores in farmers that practiced extensive and semi-extensive pig husbandry when compared to those that practiced intensive pig husbandry (40). In the present study, the higher biosecurity score observed in the intensive system (corralled pigs) could be due to the fact that good biosecurity practices are more easily implemented in confined husbandry systems and more difficult to implement when pigs are tethered or free-range (23). The findings that showed farmers who raised exotic breed or mixed breed pigs had higher biosecurity scores when compared to those that raised local breed pigs is expected and should not be surprising. In Uganda, the majority of farmers raise exotic/mixed breed pigs in confinement where biosecurity is more implementable because they are perceived to be more susceptible to diseases and environmental stress, and are economically more valuable while local breed pigs are raised under the free-range or extensive/semi-intensive husbandry system (28).

In the present study, many farmer respondents said they were knowledgeable about ASF, 43% (41/96) reported having ASF sick pigs experienced ASF outbreaks in the past 12 months, with mortalities reported by 85% of the 41 farmers. Comparing our findings to previous studies, a survey conducted in Masaka and Rakai districts of Uganda found that most pig farmers were aware of ASF, and 21% (51/541) had experienced ASF outbreaks 1 to 2 years prior to the onset of the survey (41). Another study conducted in 2012 through 2013 in seven districts of Uganda that are not included in the present study found 79% of 140 surveyed farmers had an ASF outbreak on their farms in 2012 (42). The reported morbidity and mortality in the present study should not be surprising because ASF is known to be endemic in Uganda (6). In the current study, we found a significant association between the level of farming experience and herd size with farmers’ reports of ASF incursions, but no significant differences in the post-hoc pairwise comparisons. The absence of significant pairwise differences post-hoc may have resulted from lack of statistical power due to the small sample size. It would be beneficial to further examine these associations using larger/more representative sample sizes.

Our findings show that animal health workers and pig buyers are the most common visitors that have contact with farmers’ pigs. By the nature of their work, animal health workers generally move from farm to farm and therefore have a high risk of spreading diseases (43). It is important that animal health workers at all levels are trained on and adhere to good biosecurity practices during their work to minimize the risk of spreading diseases. The risk of disease transmission by pig traders in Uganda is equally high when compared to animal health workers due to the nature of their trade that largely involves buying pigs directly from farms and movement from farm to farm, from village to village, and across local and regional territorial boundaries (14, 44). Therefore, training of pig buyers on good biosecurity practices may be helpful.

There are some key biosecurity improvements to consider as well. Farmers should avoid feeding pigs non-boiled swill (23) even when access to pig feeds can be a challenge. Feeding of pigs on swill was a common practice in the present study, however most farms that fed swill did not re-cook/boil it before feeding. Perhaps, this could be due to lack of awareness among farmers on the importance of boiling swill for at least 30 min before feeding it to pigs or the farmers are aware but are constrained by the financial implications associated with acquiring the necessary fuel. In a survey by Dione and others (15), farmers reported that the high cost of acquiring wood for boiling swill was a barrier to the implementation of this biosecurity measure. Our findings did not reveal any contact between the farmers’ pigs and wild pigs. This could be because farms visited were far away from protected areas and many were fenced. In response to outbreaks in the farm, we found 21% of the 99 farms surveyed sold sick pigs and key informants said this percentage was higher. Selling of sick pigs following outbreaks is an established practice in Uganda that is referred to as “panic sales” due to the panic that sets in as farmers try to avoid economic losses (5, 14, 30). At the community level, sale of sick pigs poses a huge biosecurity risk (23) and farmers need to be educated on the dangers associated with this practice. They spread disease from pig-to-pig, contaminate transport vehicles and other holding areas, and their pork is infectious (7, 45). Spread of manure from pigs was another common practice we found in the surveyed districts. At the community level, spread of manure in gardens is a high risk practice that plays a role in the dissemination of pathogens through free-ranging pigs via the fecal-oral route (23, 46). Evidence synthesis approaches have shown that ASFV can survive in contaminated environments under different conditions (47, 48). Spread of pig manure as fertilizer is inevitable, particularly as integrated livestock-crop farming systems are promoted in Uganda to enhance food security. It is therefore important that practical, and inexpensive manure treatment methods that are effective in pathogen inactivation are devised. In the meantime, farmer awareness regarding the risks associated with use of untreated or inappropriately treated manure as fertilizer may be need.

This study had some limitations. We used non-probability sampling approaches and therefore our findings may not be generalizable to the wider population of pig farmers in the selected districts. Sampling until saturation is best for identifying trends, and statistical assessments may be influenced by any selection bias introduced by this non-random sampling method. However, these methods allowed for diversity among participants leading to the inclusion of farmers of different gender, and farmers from a variety of farm sizes, locations, and pig management systems. It also allows for a survey to be developed when a sampling frame is not available and cannot be developed. Like any other survey, our findings may also be subject to self-report bias, recall bias and social desirability bias. The farmer responses are self-reported, and the farmers may have not accurately recalled past events/past practices leading to recall bias. Some respondents may have also given socially desirable responses introducing social desirability bias. However, the key strength of this study is that where possible, we triangulated the farmer responses with those from the key informants and the field observations. Overall, the findings from the field observations and the key informants survey corroborated with those from the farmer interviews.

## 5. Conclusion

Pig biosecurity is poor across the studied districts, necessitating more farmer awareness regarding the importance of good biosecurity measures through continued farmer education and access to resources to affordably implement biosecurity methods. More in-depth scientific inquiry into the enabling factors and barriers to the implementation of biosecurity practices among pig farmers in Uganda is necessary. Animal health workers and pig buyers are the most common categories of visitors that have contact with farmers pigs. Training of these categories of visitors on good biosecurity practices and their adherence to such practices could prove be beneficial for disease prevention.

## Data availability statement

The raw data supporting the conclusions of this article will be made available by the authors, without undue reservation.

## Ethics statement

This swine sampling and data collection used to identify the study districts was endorsed by the College of Veterinary Medicine, Animal Resources, and Biosecurity, Makerere University Higher Degrees Research Committee (Reference number: SBLS.EWM.2020), approved by the Uganda National Council for Science and Technology (Registration number: NS266ES). Animal work was specifically approved by Cornell University’s Institutional Animal Care and Use Committee (Protocol number: 2019-0108), and the US Army Medical Research and Development Command’s Animal Care and Use Review Office (Protocol number: CT-2020-38). Collection of pig biodata was determined to be exempt by the Cornell University’s Institutional Review Board (Protocol number: 2012010020) with a concurrence from the Human Research Protection Office of the US Army Medical Research and Development Command (Log number: A-21116). The biosecurity and supply chain survey (including the primary questionnaires and field observation checklist) was approved by Cornell University’s Institutional Review Board (Protocol number: IRB0010828) with a concurrence from the Human Research Protection Office of the US Army Medical Research and Development Command (Log number: E03438.21).

## Author contributions

KH, EW, DN, and DBN conceived the study and provided administrative oversight and support. JE, MN, DN, EW, and KH contributed to the design of the study. JE, MN, DS, KO, RK, EW, and KH collected the field data. JE, MN, DS, and KO contributed to data curation. JE conducted the statistical analyses and wrote the original draft of the manuscript. JE, MN, DS, KO, RK, DN, EW, DBN, and KH reviewed and edited the manuscript. KH, DN, and EW acquired the funding. All authors read and approved the final version of the manuscript to be published.

## Funding

This research was sponsored by the U.S. Department of the Defense, Defense Threat Reduction Agency, under grant number HDTRA1-20-1-0007. The content of the information does not necessarily reflect the position or the policy of the federal government, and no official endorsement should be inferred.

## Conflict of interest

The authors declare that the research was conducted in the absence of any commercial or financial relationships that could be construed as a potential conflict of interest.

## Publisher’s note

All claims expressed in this article are solely those of the authors and do not necessarily represent those of their affiliated organizations, or those of the publisher, the editors and the reviewers. Any product that may be evaluated in this article, or claim that may be made by its manufacturer, is not guaranteed or endorsed by the publisher.
