# Supplementary material for: A survey of biosecurity practices of pig farmers in selected districts affected by African swine fever in Uganda
Source: Front Vet Sci. 2023 Aug 17;10:1245754. doi: 10.3389/fvets.2023.1245754 (PMC10469975; doi:10.3389/fvets.2023.1245754)
Supplement: Supplementary file 1 [file Data_Sheet_1.zip › Supplementary file 1_Farmer questionnaire in English.pdf]

Biosecurity practices of pig farmers in African swine fever hotspots in Uganda

Informed consent

After the informed consent briefing, did the farmer give his or her consent to participate?

- ◯ Yes
- ◯ No

Part 1

Interview date

Interviewer initials e.g. KH for Karyn Havas

Interview ID e.g. Interview 1 for the first interview

» Interviewer fills out – do not ask

Gender of the respondent

- ◯ Male
- ◯ Female

District where the piggery or homestead is located

County where the piggery or homestead is located

Subcounty where the piggery or homestead is located

Local council 1 village where the piggery or homestead is located

» Interview begins here

We thank you again for accepting to participate in our study. Our goal today is to complete a questionnaire with you in the shortest time possible. Some questions may appear repetitive, but we are asking them so that we can understand different ways you prevent and control African swine fever and where you buy and sell your pigs. During the interview we request that we focus on answering the interview questions. If you have other items to discuss or questions, we are happy to talk more after we have ended the interview. During the interview we will read to you the question and all the available answer options from which you can choose from.

I am going to ask you some general questions regarding your pigs.

Name up to 3 reasons that you raise pigs?

How many years has your household kept pigs?

What is the pig husbandry (pig raising) system that you use? Select all that apply

- [ ] Free-range/scavenging in the village (extensive system)
- [ ] Confinement in corrals (intensive system)
- [ ] Tethering (semi-extensive/extensive system)

Do you fence in your pigs to prevent other animals or pets from entering?

- ○ No
- ○ Yes

In a normal year, how many pigs do you keep on average?

- ○ 1-3 pigs
- ○ 4-11 pigs
- ○ 12-20 pigs
- ○ 21-30 pigs
- ○ 31-40 pigs
- ○ 41-50 pigs
- ○ > 50 pigs

What breed of pigs do you keep? Select all that apply

- [ ] Local
- [ ] Mixed
- [ ] Exotic

How many of each type of pig that I name do you keep each year on average?

Adult female pigs used for mating

Weaned piglets (Piglets that are no longer suckling)

Nursing piglets (suckling piglets)

Adult males used for mating (boars)

What is the source of labor for your pigs?

- ○ Family
- ○ Externally employed persons
- ○ Both family and externally employed persons
- ○ Other

Specify the other source of labor for your pigs

What are the common diseases that affect your pigs?

Do you know a disease of pigs called African swine fever?

A local name for African swine fever will be mentioned by the interviewer if available

- ○ Yes
- ○ No

How does a pig that has African swine fever look like?

What do you know about how pigs get African swine fever?

Where did you first hear about African swine fever?

The next question asks about African swine fever in the form I am about to describe. It is a disease that only infects pigs. It can make almost all your pigs very sick, and many, if not all, will die. They will stop eating, become feverish, get very red skin and purple ears, and they may develop diarrhea and respiratory problems before they die.

In the past 12 months, do you think your pigs became sick with African swine fever?

- Yes
- No
- Not sure

How many became sick?

In the past 12 months, do you think any of your pigs died of African swine fever?

- Yes
- No
- Not sure

How many died?

Part 2

In this part, I am going to ask you questions that are specific to the type of pig production system that you have.

What type of production system do you have? Select all that apply

- ☐ Farrow to Finish - pigs farrow on your farm and you raise them until they are sold for meat
- ☐ Breeding sows and gilts (adult female pigs) - pigs farrow on your farm and you sell weaned pigs to others to raise
- ☐ Boar service - you keep a boar (mating males) to sell breeding services
- ☐ Wean to finish/grower – you buy weaned pigs and grow them until they are sold for meat

Where do you obtain your pigs?	All born on the farm/homestead	Some are born on the farm/homestead, and some are obtained from outside sources	All are obtained from outside sources
Adult female pigs used for mating	◯ No ◯ Yes	◯ No ◯ Yes	◯ No ◯ Yes
Boars used to sell breeding services	◯ No ◯ Yes	◯ No ◯ Yes	◯ No ◯ Yes
Weaned pigs	◯ No ◯ Yes	◯ No ◯ Yes	◯ No ◯ Yes

What are the outside sources of your pigs? Select all that apply	Purchased at a livestock market	Purchased from fellow farmers	Given by a project/NGO	Other source

**Adult female pigs used for mating**	◯ Yes ◯ No	◯ Yes ◯ No	◯ No ◯ Yes	
**Boars used to sell breeding services**	◯ Yes ◯ No	◯ Yes ◯ No	◯ No ◯ Yes	
**Weaned pigs**	◯ Yes ◯ No	◯ Yes ◯ No	◯ No ◯ Yes	

**From which districts do you obtain your pigs**	**For each category of pigs, list the district(s) mentioned in the space provided**
**Adult female pigs used for mating**	
**Boars used to sell breeding services**	
**Weaned pigs**	

**How do you determine the health of the pigs before you obtain them? Select all that apply**	**An animal health worker examines the pigs**	**You examine the pigs**	**My fellow farmer(s) examine the pigs**	**I do not determine the health of the pigs before obtaining**	**Other**

**Adult female pigs used for mating**	○ No ○ Yes	○ No ○ Yes	○ No ○ Yes	○ No ○ Yes	
**Boars used to sell breeding services**	○ No ○ Yes	○ No ○ Yes	○ No ○ Yes	○ No ○ Yes	
**Weaned pigs**	○ No ○ Yes	○ No ○ Yes	○ No ○ Yes	○ No ○ Yes	

**How are the pigs transported to you once acquired?**	**For each category of pigs, list the transportation means mentioned in the space provided**
**Adult female pigs used for mating**	
**Boars used to sell breeding services**	
**Weaned pigs**	
**If you use a vehicle, is it shared with others that move pigs with it? A vehicle means a thing used for transporting pigs such as a car, truck, motorcycle, bicycle, wheelbarrow etc.**	**For each category of pigs, select the most appropriate option**

**Adult female pigs used for mating**	◯ Almost all of the time ◯ Sometimes ◯ Rarely ◯ No ◯ Not applicable
**Boars used to sell breeding services**	◯ Almost all of the time ◯ Sometimes ◯ Rarely ◯ No ◯ Not applicable
**Weaned pigs**	◯ Almost all of the time ◯ Sometimes ◯ Rarely ◯ No ◯ Not applicable
**In a normal year, how many times do you obtain pigs?**	**For each category of pigs, enter the respondent's answer in the space provided**
**Adult female pigs used for mating**	
**Boars used to sell breeding services**	
**Weaned pigs**	

» Questions for farmers who do farrow to finish and/or breed sows and gilts

How do you breed your female pigs? Select all that apply

- [ ] Uses a boar
- [ ] Uses artificial insemination

» » Uses a boar

Where do you source your boars?

- ◯ All our boars are born on the farm and kept
- ◯ Some are born on the farm/homestead, and some are obtained from outside sources
- ◯ All are obtained from outside sources

» » » Questions for farmers whose boars are obtained from outside sources

What are your outside sources of boars? Select all that apply

- [ ] Use our own boar purchased a boar at a livestock market
- [ ] Use our own boar given to you to own by a project/NGO
- [ ] Service given by project/NGO
- [ ] Pay for boar service-communal service
- [ ] Pay for boar service-private service
- [ ] Use a boar as a favor from a neighbor
- [ ] Other source

Specify your other outside source(s) of boars

From which districts do you obtain boar services or boars themselves?

How do you determine the health of the boars you obtain or that mate with your other pigs? Select all that apply

- [ ] An animal health worker examines the pigs
- [ ] The farmer examines the pigs
- [ ] My fellow farmers examine the pigs
- [ ] I do not determine the health of the boars
- [ ] Other

Specify the other ways that you use to determine the health of the boars you obtain or that mate with your other pigs

How are the boars transported to you once acquired?

If you use a vehicle, is it shared with others that move pigs with it? A vehicle means a thing used for transporting pigs such as a car, truck, motorcycle, bicycle, wheelbarrow etc.

- Almost all the time
- Sometimes
- Rarely
- No
- Not applicable

How many times in a normal year are BOARS OF OTHERS used to mate with your adult female pigs?

How many times in a normal year do you use YOUR BOARS to mate with your adult female pigs?

» » Uses artificial insemination

What is the main source of the semen for your artificial insemination?

Is the semen tested for any diseases before it is used?

- No
- Yes
- Not sure

What diseases?

In a normal year, how many times do you inseminate your pigs?

How many times in a normal year do your adult female pigs farrow?

» Questions for farmers involved in farrow to finish

What types of pigs do you sell? Select all that apply

- [ ] Female pigs for breeding
- [ ] Recently weaned pigs to others to raise
- [ ] Full-grown pigs for slaughter
- [ ] Boars used for breeding

For each type of pig that I will mention to you, mention whom you sell or give them to

....

Female pigs for breeding. Select all that apply

- [ ] Trader
- [ ] Butcher
- [ ] Other farmers
- [ ] Family
- [ ] Buyers at markets
- [ ] Other

Specify other persons to whom you sell or give your female pigs

....

Average number sold or given away in a normal year

....

To which districts are they sold to?

....

Recently weaned piglets for others to raise. Select all that apply

- [ ] Trader
- [ ] Butcher
- [ ] Other farmers
- [ ] Family
- [ ] Buyers at markets
- [ ] Other

Specify other persons to whom you sell or give your weaned piglets

Average number sold or given away in a normal year

To which districts are they sold or given to?

Full-grown pigs for slaughter. Select all that apply

- [ ] Trader
- [ ] Butcher
- [ ] Other farmers
- [ ] Family
- [ ] Buyers at markets
- [ ] Local slaughter slab
- [ ] Other slaughter

Where is the local slaughter slab?

Where does the other slaughter take place?

Average number sold or given away in a normal year

To which districts are they sold to?

Boars. Select all that apply

- [ ] Trader
- [ ] Butcher
- [ ] Other farmers
- [ ] Family
- [ ] Buyers at markets
- [ ] Other

Specify other persons to whom you sell or give boars

Average number sold or given away in a normal year

To which districts are they sold or given to?

» Questions for farmers involved in breeding to sell weaned pigs

How many female pigs born on your farm/homestead do you keep and raise for breeding in a normal year?

What types of pigs do you sell? Select all that apply

- [ ] Female pigs for breeding
- [ ] Recently weaned pigs to others to raise
- [ ] Boars used for breeding

For each type of pig that I mention to you below, mention who you sell or give them to

Female pigs for breeding. Select all that apply

- [ ] Trader
- [ ] Butcher
- [ ] Other farmers
- [ ] Family
- [ ] Buyers at markets
- [ ] Other

Specify the other persons to whom you sell or give your adult female pigs

Average number sold or given away in a normal year

To which districts are they sold to?

Recently weaned piglets. Select all that apply

- Trader
- Butcher
- Other farmers
- Family
- Buyers at markets
- Other

Specify the other persons to whom you sell or give your weaned piglets

Average number sold or given away in a normal year

To which districts are they sold to?

Boars for breeding. Select all that apply

- Trader
- Butcher
- Other farmers
- Family
- Buyers at markets
- Other

Specify the other persons to whom you sell or give your boars for breeding

Average number sold or given away in a normal year

To which districts are they sold to?

» Questions for farmers involved in boar service

How many times in a normal year do you keep boars that are born on your farm and use them for boar service?

Who do you sell or give your boars to? Select all that apply

- [ ] Trader
- [ ] Butcher
- [ ] Other farmers
- [ ] Family
- [ ] Buyers at markets
- [ ] Local slaughter slab
- [ ] Other slaughter

Where is the local slaughter slab?

Where does the other slaughter take place?

Average number sold or given away in a normal year

To which districts are they sold to?

Who uses your boars to breed their female pigs? Select all that apply

- [ ] Neighbors
- [ ] Other villagers
- [ ] Family
- [ ] Other

Specify other

How often do they use your boars to breed their adult pigs in a normal year?

In which districts?

» Questions for farmers involved in wean to finish pig production

How many times in a normal year do you get weaned and/or grower pigs?

Who do you sell or give your full-grown pigs to? Select all that apply

- [ ] Trader
- [ ] Butcher
- [ ] Other farmers
- [ ] Family
- [ ] Local slaughter slab
- [ ] Other slaughter

Where is the local slaughter slab?

Where does the other slaughter take place?

Average number sold or given away in a normal year

In which districts are they sold?

Part 3

In this part, I am going to ask you questions that pertain to all pigs that are newly introduced.

Are new animals quarantined before their introduction to your other pigs?

Quarantine means newly brought animals are separated from the other animals for a period of time, and separation means the newly brought pigs do not have any contact with your other pigs

- Almost all of the time
- Sometimes
- Rarely
- No
- Not sure

Where do you quarantine new animals?

Where do you quarantine new animals?

Where do you quarantine new animals?

For how long are new animals quarantined?

For how long are new animals quarantined?

For how long are new animals quarantined?

What would prevent you from taking the animals out of quarantine?

What would prevent you from taking the animals out of quarantine?

What would prevent you from taking the animals out of quarantine?

How do you introduce new animals into the piggery or homestead?

Are unsold animals brought back from the market and kept with your other pigs?

- ◯ Almost all of the time
- ◯ Sometimes
- ◯ Rarely
- ◯ No
- ◯ Not applicable

Part 4

In this part, I am going to ask you questions that pertain to your farm/piggery/pig enterprise as a whole.

Does anyone who has contact with your pigs at the farm/household have contact with other pigs?

- ◯ Almost all of the time
- ◯ Sometimes
- ◯ Rarely
- ◯ No
- ◯ Not sure

What do you feed your pigs? Select all that apply

- ☐ Household leftovers
- ☐ Restaurant waste/leftovers from parties or other functions
- ☐ Commercially bought feed
- ☐ Pasture
- ☐ Crop residues
- ☐ Other sources

Specify your other sources of feeds for your pigs

If other, are meat scraps in it?

- ◯ Almost all of the time
- ◯ Sometimes
- ◯ Rarely
- ◯ No
- ◯ Not sure

If household leftovers, are meat scraps ever found in the household leftovers?

- Almost all of the time
- Sometimes
- Rarely
- No
- Not sure

If restaurant waste/leftovers from parties or other functions, are meat scraps ever found in restaurant waste?

- Almost all of the time
- Sometimes
- Rarely
- No
- Not sure

If household leftovers, are these cooked or boiled again before feeding to pigs?

- No
- Rarely
- Sometimes
- Almost all of the time

If restaurant waste/leftovers from parties or other functions, are these cooked or boiled again before feeding to pigs?

- No
- Rarely
- Sometimes
- Almost all of the time

For the next few questions, we are going to ask about visitors. A visitor means anyone who doesn't provide daily care to the pigs or are not members of the household and these may include relatives, animal health workers, neighbors etc.

Do visitors ever go into the area where pigs are kept?

- ◯ Almost all of the time
- ◯ Sometimes
- ◯ Rarely
- ◯ No

» Questions related to visitors

Which people come in contact with your pigs? Select all that apply

- ☐ Neighbors
- ☐ Animal health workers
- ☐ Community leaders
- ☐ Family members from outside the household
- ☐ Pig buyers
- ☐ Others

Specify other people who come into contact with your pigs

Are footbaths with disinfectant routinely present at the entrance to the pig housing?

- ○ No
- ○ Rarely
- ○ Sometimes
- ○ Almost all of the time
- ○ Not applicable

Do you provide all visitors any farm specific clothes and footwear when they contact your pigs?

- ○ No
- ○ Rarely
- ○ Sometimes
- ○ Almost all of the time

Do they clean their footwear before coming into contact with your pigs?

- ○ No
- ○ Rarely
- ○ Sometimes
- ○ Almost all of the time

Do you control for flies around your pigs?

- ◯ No control is done
- ◯ Rarely
- ◯ Sometimes
- ◯ Almost all of the time

Do you control for rodents around your pigs?

- ◯ No control is done
- ◯ Rarely
- ◯ Sometimes
- ◯ Almost all the time

Do dogs have direct contact with your pigs?

- ◯ Almost all of the time
- ◯ Sometimes
- ◯ Rarely
- ◯ No
- ◯ Not sure

Do cats have direct contact with your pigs?

- ◯ Almost all the time
- ◯ Sometimes
- ◯ Rarely
- ◯ No
- ◯ Not sure

Now we are going to ask you if other animals have contact with your pigs

Do any other livestock have direct contact with your pigs?

- ◯ Almost all of the time
- ◯ Sometimes
- ◯ Rarely
- ◯ No
- ◯ Not sure

Do poultry have direct contact with your pigs?

- ○ Almost all the time
- ○ Sometimes
- ○ Rarely
- ○ No
- ○ Not sure

Do your pigs mix with those of other farmers in the neighborhood?

- ○ Almost all of the time
- ○ Sometimes
- ○ Rarely
- ○ No

Why does this happen?

....

In the past 12 months, have you seen wild pigs such as warthogs or bushpigs around your homestead or village?

- ○ Yes
- ○ No

In the past 12 months, have you seen these wild pigs contact your pigs?

- ○ Yes
- ○ No
- ○ Not sure

What do you do with sick pigs? Select all that apply

- ☐ You isolate sick pigs from healthy ones
- ☐ You treat the sick
- ☐ You sell off the sick
- ☐ You slaughter and consume
- ☐ You slaughter and sell the meat
- ☐ Other

Specify the other way(s) you deal with sick pigs

....

We will now ask you what your household and employees do before working with pigs

Do you wash your hands before working with pigs?

- Almost all of the time
- Sometimes
- Rarely
- No

Do you use clothing dedicated to working with your pigs?

- Almost all of the time
- Sometimes
- Rarely
- No

Do you use footwear dedicated to working with your pigs?

- No
- Rarely
- Sometimes
- Almost all of the time

Do you avoid contact with your pigs for at least two days after coming in contact with other people's pigs?

- No
- Rarely
- Sometimes
- Almost all of the time

Is the pig pen/holding area regularly cleaned?

- Yes
- No

How often do you clean your pig pen/holding area?

- Daily
- Weekly
- Monthly
- Other

Specify how often you clean your pig pen/holding area

How do you clean your pig pen/holding area?

Is any equipment that you use shared with other pig farmers?

Equipment includes veterinary medical equipment, tools, and any production equipment.

- Yes
- No

Is it cleaned and disinfected between farms?

- Yes
- No
- Not sure

What do you do with the manure from your pigs?

We now ask you a few questions about how you dispose of pigs that die in your piggery/household

Do you bury them?

- No
- Rarely
- Sometimes
- Almost all of the time

Do you burn them?

- No
- Rarely
- Sometimes
- Almost all of the time

Do you feed them to dogs?

- No
- Rarely
- Sometimes
- Almost all of the time

Do you sell pork from the dead pigs?

- No
- Rarely
- Sometimes
- Almost all of the time

Are dead pigs thrown to the bushes?

- No
- Rarely
- Sometimes
- Almost all of the time

What else do you do with dead pigs? (Specify)

....

List up to THREE difficulties that you face when attempting to control diseases in your pigs

....

List up to THREE of your greatest strengths in disease control

....

What ADDITIONAL information or comments would you like to provide regarding what we have talked about during this interview?

....

Interviewer: Please remember to thank the farmer for participating in this survey.

Interviewer: Please thank the farmer for their time and exit!

Interviewer: Please remember to give the respondent an incentive of UGX 20,000.
