# Supplementary material for: A survey of biosecurity practices of pig farmers in selected districts affected by African swine fever in Uganda
Source: Front Vet Sci. 2023 Aug 17;10:1245754. doi: 10.3389/fvets.2023.1245754 (PMC10469975; doi:10.3389/fvets.2023.1245754)
Supplement: Supplementary file 1 [file Data_Sheet_1.zip › Supplementary file 2_Key informants' questionnaire.pdf]

Biosecurity practices of pig farmers in African swine fever hotspots in Uganda

---

Start of Block: Introduction and informed consent statement

Q1 In this study, we seek to document the opinions of subject matter experts such as veterinarians and researchers on farmer biosecurity practices at African swine fever hotspots in Uganda, and to identify where the pig farmers buy and sell their stock. For this survey, there is no back button because of the skip logic utilized. Therefore, you will not be able to move back to change your responses.

Q2 Please click and read the informed consent statement provided below.
Informed consent statement I have read and understood the information in the attached informed consent statement. Do you agree to participate in this survey?

- ○ Yes (1)
- ○ No (2)

Skip To: End of Survey If Please click and read the informed consent statement provided below. Informed consent statement I... = No

End of Block: Introduction and informed consent statement

Start of Block: General questions

Q3 Where do you work?

- ◯ Central government (Ministry of Agriculture, Animal Industry and Fisheries) (1)
- ◯ Academia (Mention institution) (2) ____
- ◯ Non-Governmental Organization (NGO) (Specify) (3) ____
- ◯ Local government (4)
- ◯ Private sector (Specify) (5) ____
- ◯ Other (Specify) (6) ____

Display This Question:

If Where do you work? = Non-Governmental Organization (NGO) (Specify)

Q4 In which district(s) of Uganda do you work?

____

Display This Question:

If Where do you work? = Local government

Q5 In which district do you work?

____

Display This Question:

If Where do you work? = Local government

Q6 In which county do you work?

____

Display This Question:

If Where do you work? = Local government

Q7 In which subcounty do you work?

____

Q8 Which of the following best describes your professional role?

- ◯ Veterinarian (1)
- ◯ Para-veterinarian (2)
- ◯ Animal husbandry officer (3)
- ◯ Animal scientist (4)
- ◯ Other (specify) (5) ____

Q9 Which of the following best describes your level of knowledge about pig farmers' biosecurity practices in central Uganda?

- ◯ Not knowledgeable (1)
- ◯ Slightly knowledgeable (2)
- ◯ Moderately knowledgeable (3)
- ◯ Very Knowledgeable (4)
- ◯ Extremely knowledgeable (5)

Skip To: End of Survey If Which of the following best describes your level of knowledge about pig farmers' biosecurity prac... = Not knowledgeable

Q10 Name up to 3 reasons why farmers you interact with raise pigs

____

____

____

____

____

Q11 What is the **predominant** pig husbandry system for the farmers you interact with in central Uganda?

- ○ Free-range/scavenging in the village (extensive system) (1)
- ○ Confinement in corrals (intensive system) (2)
- ○ Tethering (semi-extensive/extensive system) (3)
- ○ None are predominant (4)
- ○ I don’t know (5)

Q12 Rank the following production systems based on how common they are among the farmers you interact with.

	I don't know (1)	Not common (2)	Common (3)	Very common (4)
Farrow to Finish (1)	○	○	○	○
Breeding sows and gilts (2)	○	○	○	○
Boar service (3)	○	○	○	○
Wean to finish/grower (4)	○	○	○	○
Other (specify (5)	○	○	○	○

End of Block: General questions

Start of Block: Biosecurity

Q13 For questions in this section, we define biosecurity as the practices, tools, and/or methods farmers use to prevent disease in their pigs.

Q14 Rate how common the following methods are for farmers to evaluate the health of animals brought to their farm/household.

	I don't know (1)	Not common (2)	Common (3)	Very common (4)
An animal health worker examines the pigs (1)	○	○	○	○
The farmer examines the pigs (2)	○	○	○	○
Other farmers examine the pigs (3)	○	○	○	○
Other (specify) (4)	○	○	○	○

Q15 What percentage of the farmers that you interact with have fences around their pigs to prevent other animals from entering?

- None (1)
- 1-25% (2)
- 26-50% (3)
- 51-75% (4)
- 76-100% (5)
- I do not know (6)

Q16 In your opinion, what proportion of pig farmers that you interact with quarantine new animals before their introduction to other pigs? (Quarantine means newly brought animals are separated from the other animals for a period of time).

- None (1)
- 1-25% (2)
- 26-50% (3)
- 51-75% (4)
- 76-100% (5)
- I do not know (6)

Skip To: Q19 If In your opinion, what proportion of pig farmers that you interact with quarantine new animals bef... = None

Skip To: Q20 If In your opinion, what proportion of pig farmers that you interact with quarantine new animals bef... = I do not know

---

Q17 For how long are new animals quarantined by an average farmer?

____

Q18 What percentage of farmers evaluate their pigs before taking them out of quarantine?

- ○ None (1)
- ○ 1-25% (2)
- ○ 26-50% (3)
- ○ 51-75% (4)
- ○ 76-100% (5)
- ○ I do not know (6)

Q19 How do farmers introduce new animals into their piggeries or homesteads if they do not quarantine?

____

____

____

____

____

Q20 What percentage of pig farmers return unsold animals to their farms/homesteads from the market and keep them with other pigs?

- None (1)
- 1-25% (2)
- 26-50% (3)
- 51-75% (4)
- 76-100% (5)
- I do not know (6)

Q21
Rate how common it is for pig farmers to use the following as feed for their pigs.

	I don't know (1)	Not common (2)	Common (3)	Very common (4)
Household wastes (leftover human food or waste from food preparation) (1)	○	○	○	○
Restaurant wastes/leftovers from parties and/or other functions (2)	○	○	○	○
Commercially bought feed (3)	○	○	○	○
Pasture (4)	○	○	○	○
Crop residues (e.g. plants not used for human consumption) (5)	○	○	○	○
Other sources (specify) (6)	○	○	○	○

Q22 What percentage of farmers use household leftovers, restaurant waste, leftovers from parties or other social functions that contain meat scraps as pig food?

- None (1)
- 1-25% (2)
- 26-50% (3)
- 51-75% (4)
- 76-100% (5)
- I do not know (6)

Skip To: Q24 If What percentage of farmers use household leftovers, restaurant waste, leftovers from parties or o... = None

Skip To: Q24 If What percentage of farmers use household leftovers, restaurant waste, leftovers from parties or o... = I do not know

Q23 For use of household leftovers, restaurant waste, leftovers from parties or other social functions as pig food, what percentage of farmers cook or boil the waste before feeding it to pigs?

- None (1)
- 1-25% (2)
- 26-50% (3)
- 51-75% (4)
- 76-100% (5)
- I do not know (6)

Q24 What percentage of farmers routinely provide foot baths with disinfectant at the entrance to their pig housing?

- None (1)
- 1-25% (2)
- 26-50% (3)
- 51-75% (4)
- 76-100% (5)
- I do not know (6)

Q25 For the next few questions, we are asking about visitors. A visitor is anyone who doesn’t live in the household or provide daily care to the pigs. Visitors may include relatives, animal health workers etc.

Q26 What percentage of farmers let people who have contact with pigs from other farms/households contact their pigs?

- None (1)
- 1-25% (2)
- 26-50% (3)
- 51-75% (4)
- 76-100% (5)
- I do not know (6)

Q27 Rank the following categories of visitors based on how commonly they are allowed to go into the areas where pigs are kept.

	I don't know (1)	Never allowed (2)	Least commonly allowed (3)	Commonly allowed (4)	Most commonly allowed (5)
Neighbors (1)	○	○	○	○	○
Animal health workers (2)	○	○	○	○	○
Community leaders (3)	○	○	○	○	○
Family (4)	○	○	○	○	○
Pig buyers (5)	○	○	○	○	○
Other (specify) (6)	○	○	○	○	○
Other (specify) (7)	○	○	○	○	○

Q28 What percentage of pig farmers provide visitors with farm specific clothes and/or footwear when they contact pigs?

○ None (1)

○ 1-25% (2)

○ 26-50% (3)

○ 51-75% (4)

○ 76-100% (5)

○ I do not know (6)

Display This Question:

If What percentage of pig farmers provide visitors with farm specific clothes and/or footwear when t... = None

Q29 If footwear is not provided, what percentage of visitors clean their footwear before contacting the pigs?

- None (1)
- 1-25% (2)
- 26-50% (3)
- 51-75% (4)
- 76-100% (5)
- I do not know (6)

Q30 What percentage of pig farmers control flies around their pigs?

- None (1)
- 1-25% (2)
- 26-50% (3)
- 51-75% (4)
- 76-100% (5)
- I do not know (6)

Q31 What percentage of pig farmers control rodents around their pigs?

- None (1)
- 1-25% (2)
- 26-50% (3)
- 51-75% (4)
- 76-100% (5)
- I do not know (6)

Q32 What percentage of household pigs have contact with village dogs?

- None (1)
- 1-25% (2)
- 26-50% (3)
- 51-75% (4)
- 76-100% (5)
- I do not know (6)

Q33 What percentage of household pigs have contact with cats?

- None (1)
- 1-25% (2)
- 26-50% (3)
- 51-75% (4)
- 76-100% (5)
- I do not know (6)

Q34 What percentage of farmers have pigs that come in contact with other livestock?

- None (1)
- 1-25% (2)
- 26-50% (3)
- 51-75% (4)
- 76-100% (5)
- I do not know (6)

Q35 What percentage of farmers have pigs that come in contact with poultry?

- ○ None (1)
- ○ 1-25% (2)
- ○ 26-50% (3)
- ○ 51-75% (4)
- ○ 76-100% (5)
- ○ I do not know (6)

Q36 What percentage of farmers have pigs that come in regular contact with pigs of other farmers?

- ○ None (1)
- ○ 1-25% (2)
- ○ 26-50% (3)
- ○ 51-75% (4)
- ○ 76-100% (5)
- ○ I do not know (6)

Q37 What percentage of pig farmers report that wild pigs, such as warthogs or bush pigs, roam around their homesteads or villages?

- None (1)
- 1-25% (2)
- 26-50% (3)
- 51-75% (4)
- 76-100% (5)
- I do not know (6)

Skip To: Q39 If What percentage of pig farmers report that wild pigs, such as warthogs or bush pigs, roam around... = None

Q38 What percentage of farmers report that wild pigs come into contact with their pigs?

- None (1)
- 1-25% (2)
- 26-50% (3)
- 51-75% (4)
- 76-100% (5)
- I do not know (6)

Q39 Rank how common the following practices are regarding what pig farmers do with sick pigs.

	I do not know (1)	Not common (2)	Common (3)	Very common (4)
Isolate sick ones from healthy ones (1)	○	○	○	○
Treat the sick (2)	○	○	○	○
Sell off the sick pigs (3)	○	○	○	○
Slaughter and consume (4)	○	○	○	○
Slaughter and sell the meat (5)	○	○	○	○
Other (specify) (6)	○	○	○	○

Q40 Which of the following best represent common practices of pig farmers before working with pigs?

	I do not know (1)	Not common (2)	Common (3)	Very common (4)
Wash hands (1)	○	○	○	○
Put on clean clothes (2)	○	○	○	○
Put on clean footwear (3)	○	○	○	○
Other measure taken (specify) (4)	○	○	○	○
Other measure taken (specify) (5)	○	○	○	○

Q41 In your opinion, what percentage of pig farmers clean their pig pens or holding area?

- ○ None (1)
- ○ 1-25% (2)
- ○ 26-50% (3)
- ○ 51-75% (4)
- ○ 76-100% (5)
- ○ I don't know (6)

Skip To: Q44 If In your opinion, what percentage of pig farmers clean their pig pens or holding area? = None

Skip To: Q44 If In your opinion, what percentage of pig farmers clean their pig pens or holding area? = I don't know

Q42 On average, how often do they clean the pig pens?

____

Q43 Briefly describe how pig pens are cleaned.

____

____

____

____

____

Q44 What percentage of pig farmers commonly share equipment with other pig farmers? Equipment includes medical equipment, tools, and any production equipment.

- None (1)
- 1-25% (2)
- 26-50% (3)
- 51-75% (4)
- 76-100% (5)
- I do not know (6)

Skip To: Q46 If What percentage of pig farmers commonly share equipment with other pig farmers? Equipment include... = None

Skip To: Q46 If What percentage of pig farmers commonly share equipment with other pig farmers? Equipment include... = I do not know

---

Q45 What percentage of pig farmers clean and disinfect equipment between farms?

- None (1)
- 1-25% (2)
- 26-50% (3)
- 51-75% (4)
- 76-100% (5)
- I do not know (6)

Q46 What do pig farmers do with the manure from their pigs?

____

____

____

Q47 Rank how common the following practices are regarding how pig farmers dispose of dead pigs.

	I do not know (1)	Not at all common (2)	Slightly common (3)	Moderately common (4)	Very common (5)	Extremely common (6)
Buried (1)	○	○	○	○	○	○
Burned (2)	○	○	○	○	○	○
Fed to dogs (3)	○	○	○	○	○	○
Eaten at home (4)	○	○	○	○	○	○
Sell the meat (5)	○	○	○	○	○	○
Thrown to the bushes (6)	○	○	○	○	○	○
Other (specify) (7)	○	○	○	○	○	○

Q48 What do you think are farmers' greatest strengths in disease control?

Q49 What are the challenges that pig farmers face in disease control?

____

____

____

____

____

End of Block: Biosecurity

Start of Block: Supply chain

Q50 Rate how common it is for farmers to obtain adult female pigs (sows and/or gilts) from the sources listed below. These are pigs that are used for breeding by pig farmers.

	I do not know (1)	Not common (2)	Common (3)	Very common (4)
Born on the farm (1)	o	o	o	o
Livestock market (2)	o	o	o	o
From fellow farmers (3)	o	o	o	o
Given by a project/NGO (4)	o	o	o	o
Other source (specify) (5)	o	o	o	o

Q51 Rate how common it is for farmers to obtain weaned and/or growing pigs from the sources listed below. These are pigs that are raised to be slaughtered.

	I do not know (1)	Not common (2)	Common (3)	Very common (4)
Livestock market (1)	○	○	○	○
From fellow farmers in the village (2)	○	○	○	○
Born on the farm (3)	○	○	○	○
Given by a project/NGO (4)	○	○	○	○
Other source (specify) (5)	○	○	○	○

Q52 Rate how common it is for farmers to obtain boars for breeding from the sources listed below. These are pigs that are used to breed female pigs.

	I do not know (1)	Not common source (2)	Common (3)	Very common source (4)
Born on the farm (1)	○	○	○	○
Livestock market (2)	○	○	○	○
Given by a project/NGO (3)	○	○	○	○
Imported from other country (5)	○	○	○	○
Other (specify) (4)	○	○	○	○

Q53 Where do farmers get their boar service from? A boar service is when a boar breeds a female pig. Rank the following sources based on how common they are.

	I don't know (1)	Not a common source (2)	Common source (3)	Very common source (4)
Use their own boar (1)	○	○	○	○
Pay for boar service from a communal boar service provider (2)	○	○	○	○
Pay for boar service from a private service provider (3)	○	○	○	○
Obtain boar service as a favor from neighbors (4)	○	○	○	○
Other source (specify) (5)	○	○	○	○

Q54
How do farmers transport the following pig types to their farms/households once acquired?

Select pig types you are familiar with and enter your answer in the space provided.

- ☐ Adult breeding females (4) ________________
- ☐ Breeding boars (5) ________________
- ☐ Weaned piglets (6) ________________
- ☐ Pigs for slaughter (7) ________________

Q55 If they use a vehicle, what percentage of farmers share the vehicle with others that move pigs with it at the same time? A vehicle is defined as a thing used to transport pigs, such as a car, truck, motorcycle, bicycle, wheelbarrow etc.

- ○ None (1)
- ○ 1-25% (2)
- ○ 26-50% (3)
- ○ 51-75% (4)
- ○ 76-100% (5)
- ○ I do not know (6)

Q56 Rate how common it is for farmers to sell or give their adult female pigs (sows and gilts) to each of the individuals in the list below. These are pigs that are used for breeding by pig farmers.

	I don't know (1)	Not common (2)	Common (3)	Very common (4)
Pig traders (1)	○	○	○	○
Butchers (2)	○	○	○	○
Other farmers (3)	○	○	○	○
Family (4)	○	○	○	○
Buyers at markets (5)	○	○	○	○
Other (specify) (6)	○	○	○	○

Q57 Rate how common it is for farmers to sell or give their recently weaned piglets that typically require more time to grow before being slaughtered to each of the individuals in the list below.

	I don't know (1)	Not common (2)	Common (3)	Very common (4)
Pig traders (1)	○	○	○	○
Butchers (2)	○	○	○	○
Other farmers (3)	○	○	○	○
Family (4)	○	○	○	○
Buyers at markets (5)	○	○	○	○
Other (specify) (6)	○	○	○	○

Q58 Rate how common it is for farmers to sell or give their boars to each of the individuals in the list below. These are pigs that are used to breed female pigs.

	I don't know (1)	Not common (2)	Common (3)	Very common (4)
Pig traders (1)	○	○	○	○
Butchers (2)	○	○	○	○
Other farmers (3)	○	○	○	○
Family (4)	○	○	○	○
Buyers at markets (5)	○	○	○	○
Other (specify) (6)	○	○	○	○

Q59 Rate how common it is for farmers to sell or give their full-grown pigs that are ready for slaughter to each of the individuals in the list below.

	I don't know (5)	Not common (6)	Common (7)	Very common (8)
Pig traders (1)	○	○	○	○
Butchers (2)	○	○	○	○
Other farmers (3)	○	○	○	○
Family (4)	○	○	○	○
Buyers at markets (5)	○	○	○	○
Other (specify) (6)	○	○	○	○

Q60 Do you have any other comments?

____

____

____

____

____

End of Block: Supply chain
