# Supplementary material for: A survey of biosecurity practices of pig farmers in selected districts affected by African swine fever in Uganda
Source: Front Vet Sci. 2023 Aug 17;10:1245754. doi: 10.3389/fvets.2023.1245754 (PMC10469975; doi:10.3389/fvets.2023.1245754)
Supplement: Supplementary file 1 [file Data_Sheet_1.zip › Supplementary file 3_Field observation checklist.pdf]

Field observation checklist

Interview date

Interviewer initials e.g., KH for Karyn Havas

Interview ID e.g., Interview 1 for the first interview

Observer's initials e.g., JE for John Ekakoro

Pig husbandry system

Free-range/scavenging in the village (extensive system)

- ○ Yes
- ○ No

Confinement in corrals (intensive system)

- ○ Yes
- ○ No

Tethering (semi-extensive/extensive system)

- ○ Yes
- ○ No

Breeds of pigs kept. Select all that apply

- ☐ Local
- ☐ Mixed
- ☐ Exotic

Pig feeds

- ☐ Household leftovers/ restaurant waste
- ☐ Commercially bought feed e.g., maize bran.
- ☐ Pasture
- ☐ Crop residues
- ☐ Any other

Meat scraps observed

- ( ) Yes
- ( ) No

Specify any other

____

Any farm specific clothes and footwear

- ( ) Yes
- ( ) No
- ( ) Not observed

Who works with the pigs?

____

Any visitors have contact with the pigs?

- ( ) Yes
- ( ) No
- ( ) Not observed

Flies and/or rodents around pigs

- ( ) Yes
- ( ) No
- ( ) Not observed

Dogs and/or cats mingling or in contact with pigs

- ( ) Yes
- ( ) No
- ( ) Not observed

Any other livestock or poultry mingling or in contact with pigs

- ( ) Yes
- ( ) No
- ( ) Not observed

Any wild pigs such as warthogs or bushpigs around the homestead or village?

- Yes
- No
- Not observed

Ornithodoros spp. ticks seen in the housing in which pigs are kept

- Yes
- No
- Not observed

Hand washing facility near the area where pigs are kept

- Yes
- No
- Not observed

Any clothing near the pig pens or pig keeping area that appears to be dedicated for work done in the piggery or pig keeping area

- Yes
- No
- Not observed

Any footwear such as boots seen near or found in the pig keeping area that appears to be dedicated to work on pigs

- Yes
- No
- Not observed

Pig pen/holding area appears regularly cleaned

- Yes
- No
- Not observed

Farm equipment appears clean.

- Yes
- No
- Not observed

Manure from pigs spread to nearby crop fields or gardens

- ◯ Yes
- ◯ No
- ◯ Not observed

Note any other observations related to pig biosecurity practices
