# Supplementary material for: A survey of biosecurity practices of pig farmers in selected districts affected by African swine fever in Uganda
Source: Front Vet Sci. 2023 Aug 17;10:1245754. doi: 10.3389/fvets.2023.1245754 (PMC10469975; doi:10.3389/fvets.2023.1245754)
Supplement: Supplementary file 1 [file Data_Sheet_1.zip › Supplementary file 4_Field observation checklist_Modified.pdf]

Field observation checklist_Revised on June 17, 2022

Interview date

____

Interviewer initials e.g., KH for Karyn Havas

____

Interview ID e.g., Interview 1 for the first interview

____

Observer's initials e.g., JE for John Ekakoro

____

Where you allowed to go to the pig keeping area?

- ○ Yes
- ○ No

Pig husbandry system

What is the primary pig production system?

- ☐ Free-range/scavenging in the village (extensive system)
- ☐ Confinement in corrals (intensive system)
- ☐ Tethering (semi-extensive/extensive system)
- ☐ Other production system

Specify the other production system

____

What is the secondary pig production system?

- ☐ Free-range/scavenging in the village (extensive system)
- ☐ Confinement in corrals (intensive system)
- ☐ Tethering (semi-extensive/extensive system)
- ☐ Other production system

Specify the other production system

____

If confinement, what material is the wall made of?

- [ ] Wood
- [ ] Brick and mortar-plastered
- [ ] Brick and mortar- not plastered
- [ ] Other

Specify other

If confinement, what material is the floor made of?

- [ ] Concrete
- [ ] Ground
- [ ] Indigenous microorganisms (IMO)
- [ ] Other

Specify other

Is the housing different for piglets?

Is there bedding for the pigs?

- ○ No
- ○ Yes

If yes, specify the type of bedding

Breeds of pigs kept. Select all that apply

- [ ] Local
- [ ] Mixed
- [ ] Exotic

Pig feeds

- [ ] Household leftovers/ restaurant waste (human food leftovers)
- [ ] Commercially bought feed e.g., maize bran.
- [ ] Pasture
- [ ] Crop residues (leftover plants)
- [ ] Food preparation residues (banana peels)
- [ ] Other fruits and vegetables
- [ ] Any other

Meat scraps observed

- ○ Yes
- ○ No

Specify any other

____

Who works with the pigs?

____

Any visitors have contact with the pigs?

- ○ Yes
- ○ No
- ○ Not observed

What is the proximity of the pigs to the living area?

- ○ Designated pig area very close to the living area
- ○ Designated pig area is on the property but semi-removed from the living area
- ○ Designated pig area is far way from the living area

Is there evidence of fly and/or rodent control?

- ○ Yes
- ○ No

Are there dogs and/or cats present on the farm?

- ○ Yes
- ○ No

Is there potential for dogs and/or cats mingling or coming in contact with pigs

- ◯ Yes
- ◯ No

Is there potential for poultry mingling or coming in contact with pigs?

- ◯ Yes
- ◯ No

Is there potential for other livestock mingling or coming in contact with pigs?

- ◯ Yes
- ◯ No

Did you observe a mesh fence or wall on the property?

- ◯ Yes
- ◯ No

Does it surround the property?

- ◯ Yes
- ◯ No
- ◯ Don't know

Ornithodoros spp. ticks seen in the housing in which pigs are kept

- ◯ Yes
- ◯ No

Is there a hand washing facility near the area where pigs are kept?

- ◯ Yes
- ◯ No

Do you see any clothing near the pig pens or pig keeping area that appears to be dedicated for work done in the piggery or pig keeping area?

- ◯ Yes
- ◯ No

Do you see any footwear such as boots near the pig keeping area that appears to be dedicated to work on pigs?

- ◯ Yes
- ◯ No

Does the pig pen/holding area appear regularly cleaned?

- ◯ Yes
- ◯ No

Is the manure from pigs spread to nearby crop fields or gardens?

- ◯ Yes
- ◯ No
- ◯ Not observed

Where do they put the manure to age?

- ◯ Near the pig pen
- ◯ Far away from the pig pen
- ◯ Other

Specify other

___

Do you observe any enclosure or area that could be dedicated for quarantine of new pigs? Quarantine means no contact between new pigs and old pigs.

- ◯ Yes
- ◯ No

Any comments for quarantine?

___

Note any other observations related to pig biosecurity practices

___
