# Supplementary material for: A survey of biosecurity practices of pig farmers in selected districts affected by African swine fever in Uganda
Source: Front Vet Sci. 2023 Aug 17;10:1245754. doi: 10.3389/fvets.2023.1245754 (PMC10469975; doi:10.3389/fvets.2023.1245754)
Supplement: Supplementary file 1 [file Data_Sheet_1.zip › Supplementary tables.docx]

Supplementary Material

A survey of biosecurity practices of pig farmers in selected districts affected by African swine fever in Uganda

John E. Ekakoro*, Margaret Nawatti, David F. Singler, Krista Ochoa, Robinah Kizza, Dickson Ndoboli, Deo B Ndumu, Eddie M. Wampande and Karyn A. Havas

*** Correspondence: jekakoro@vet.k-state.edu**

**Supplementary table 1. A summary of the biosecurity items and their assigned modal weights used to score farmers biosecurity in selected districts of Uganda, June 2022 through July 2022.**

| **Biosecurity item** | **Modal score** |
| --- | --- |
| Keeping a closed herd: no introductions of any new females for breeding from outside | 10 |
| Keeping a closed herd: no introductions of any boars for mating from outside | 10 |
| Keeping a closed herd: no introduction of any weaned pigs from outside | 10 |
| Replacement females examined for health by a veterinary professional before introduction | 1 |
| Weaned pigs examined for health by a veterinary professional before introduction | 1 |
| Boars for mating are examined for health by a veterinary professional prior to introduction | 1 |
| No wild pigs seen around farmer's homestead or village | 1 |
| No wild pigs seen in contact with domestic pigs | 1 |
| Manure is not used as fertilizer in gardens | 1 |
| Newly introduced pigs quarantined | 2 |
| Farm equipment is not shared with other farmers | 3 |
| Farm equipment cleaned and disinfected between farms | 1 |
| Vehicle is not shared with others that move any type of pigs with it | 1 |
| Persons in contact with a farm's pigs do not have contact with other pigs | 2 |
| Visitors use farm specific clothes and footwear | 2 |
| Footbaths and disinfectants present | 3 |
| Isolates sick pigs | 4 |
| Treats sick pigs | 2 |
| Washes hands before working with pigs | 2 |
| Uses clothes dedicated to working with pigs | 2 |
| Uses footwear dedicated to working with pigs | 2 |
| Pig pen/holding area regularly cleaned | 3.5 |
| Control for rodents around pigs | 3 |
| Dogs do not have direct contact with farmer's pigs | 1 |

**Supplementary table 1 (continued). A summary of the biosecurity items and their assigned modal weights used to score farmers biosecurity in selected districts of Uganda, June 2022 through July 2022.**

| Cats do not have direct contact with farmer's pigs | 2 |
| --- | --- |
| Other livestock do not have direct contact with farmer's pigs | 2 |
| Poultry do not have direct contact with farmer's pigs | 2 |
| Bury dead pigs | 1.5 |
| Does not feed dead pigs to dogs | 1.5 |
| Does not throw dead pigs to the bushes | 5 |
| Visitors do not go to the area pigs are kept | 2 |
| Visitors clean their footwear before contact with pigs | 2 |
| Burns dead pigs | 1.5 |
| Does not sell pork from dead pigs | 1.5 |
| Having a fence as a physical biosecurity barrier | 10 |
| Does not sell sick pigs | 5 |
| Does not sell/consume pork from the sick pigs | 10 |
| Animals returned from the market are not kept with other pigs | 2 |
| Farmer control for flies around pigs | 1 |
| Farmer's pigs do not mix with other pigs in the neighborhood | 8 |
| Farmer does not feed pigs on household leftovers | 3 |
| Farmer does not feed pigs on restaurant leftovers | 10 |
| Meat scraps are not present in the household leftovers | 5 |
| Meat scraps are not present in the restaurant leftovers | 5 |
| Household leftovers boiled or cooked again before feeding to pigs | 10 |
| Restaurant leftovers boiled or cooked again before feeding to pigs | 10 |
| **Total score** | 169.5 |

**Supplementary table 2. The distribution of farmer responses on contact of farm pigs with other domestic animals and poultry, vermin, and pest control in selected districts of Uganda with high levels of suspect ASFV cases, June 2022 through July 2022.**

|  | **Number (%) of respondents** | | | | | |
| --- | --- | --- | --- | --- | --- | --- |
|  | **Mpigi** | **Masaka** | **Luwero** | **Kamuli** | **Wakiso** | **All districts** |
| **Biosecurity category: Contact of farm pigs with other domestic animals and poultry** | | | | | | |
| **Farmer's pigs mix with other pigs in the neighborhood** | **19 (100)** | **20 (100)** | **20 (100)** | **20 (100)** | **20 (100)** | **99 (100)** |
| No | 16 (84.2) | 19 (95) | 19 (95) | 19 (95) | 15 (75) | 88 (88.9) |
| Rarely | 1 (5.3) | 0 (0) | 0 (0) | 0 (0) | 1 (5) | 2 (2) |
| Sometimes | 2 (10.5) | 1 (5) | 1 (5) | 1 (5) | 4 (20) | 9 (9.1) |
| Almost all the time | 0 (0) | 0 (0) | 0 (0) | 0 (0) | 0 (0) | 0 (0) |
| **Dogs have direct contact with farmer's pigs** | **19 (100)** | **20 (100)** | **20 (100)** | **20 (100)** | **20 (100)** | **99 (100)** |
| No | 7 (36.8) | 8 (40) | 5 (25) | 15 (75) | 11 (55) | 46 (46.5) |
| Rarely | 2 (10.5) | 4 (20) | 2 (10) | 0 (0) | 1 (5) | 9 (9.1) |
| Sometimes | 3 (15.8) | 5 (25) | 10 (50) | 3 (15) | 5 (25) | 26 (26.3) |
| Almost all the time | 7 (36.8) | 3 (15) | 1 (5) | 1 (5) | 3 (15) | 15 (15.1) |
| Not sure | 0 (0) | 0 (0) | 2 (10) | 1 (5) | 0 (0) | 3 (3) |
| **Cats have direct contact with farmer's pigs** | **19 (100)** | **20 (100)** | **20 (100)** | **20 (100)** | **20 (100)** | **99 (100)** |
| No | 8 (42.1) | 7 (35) | 7 (35) | 10 (50) | 10 (50) | 42 (42.4) |
| Rarely | 5 (26.3) | 1 (5) | 3 (15) | 0 (0) | 1 (5) | 10 (10.1) |
| Sometimes | 1 (5.3) | 5 (25) | 3 (15) | 4 (20) | 3 (15) | 16 (16.2) |
| Almost all the time | 3 (15.8) | 1 (5) | 3 (15) | 1 (5) | 5 (25) | 13 (13.1) |
| Not sure | 2 (10.5) | 6 (30) | 4 (20) | 5 (25) | 1 (5) | 18 (18.2) |
| **Other livestock have direct contact with farmer's pigs** | **19 (100)** | **20 (100)** | **20 (100)** | **20 (100)** | **20 (100)** | **99 (100)** |
| No | 13 (68.4) | 15 (75) | 11 (55) | 15 (75) | 17 (85) | 71 (71.7) |
| Rarely | 2 (10.5) | 1 (5) | 3 (15) | 0 (0) | 1 (5) | 7 (7.1) |
| Sometimes | 1 (5.3) | 3 (15) | 3 (15) | 5 (25) | 2 (10) | 14 (14.1) |
| Almost all of the time | 2 (10.5) | 0 (0) | 0 (0) | 0 (0) | 0 (0) | 2 (2) |
| Not sure | 1 (5.3) | 1 (5) | 3 (15) | 0 (0) | 0 (0) | 5 (5.1) |
| **Poultry have direct contact with farmer's pigs** | **19 (100)** | **20 (100)** | **20 (100)** | **20 (100)** | **20 (100)** | **20 (100)** |
| No | 5 (26.3) | 3 (15) | 0 (0) | 6 (30) | 5 (25) | 19 (19.2) |
| Rarely | 2 (10.5) | 1 (5) | 1 (5) | 1 (5) | 1 (5) | 6 (6.1) |
| Sometimes | 2 (10.5) | 8 (40) | 5 (25) | 4 (20) | 4 (20) | 23 (23.2) |
| Almost all the time | 10 (52.6) | 8 (40) | 14 (70) | 9 (45) | 10 (50) | 51 (51.5) |
| **Biosecurity category: Vermin and pest control** | | | | | | |
| **Control for flies around pigs** | **19 (100)** | **20 (100)** | **20 (100)** | **20 (100)** | **20 (100)** | **99 (100)** |
| No | 9 (47.4) | 4 (20) | 5 (25) | 5 (25) | 4 (20) | 27 (27.3) |
| Rarely | 2 (10.5) | 2 (10) | 1 (5) | 0 (0) | 1 (5) | 6 (6.1) |
| Sometimes | 5 (26.3) | 10 (50) | 12 (60) | 9 (45) | 8 (40) | 44 (44.4) |
| Almost all the time | 3 (15.8) | 4 (20) | 2 (10) | 6 (30) | 7 (35) | 22 (22.2) |
| **Control for rodents around pigs** | **19 (100)** | **20 (100)** | **20 (100)** | **20 (100)** | **20 (100)** | **99 (100)** |
| No | 12 (63.2) | 7 (35) | 18 (90) | 11 (55) | 12 (60) | 60 (61) |
| Rarely | 1 (5.3) | 5 (25) | 1 (5) | 2 (10) | 3 (15) | 12 (12) |
| Sometimes | 1 (5.3) | 5 (25) | 1 (5) | 4 (20) | 3 (15) | 14 (14) |
| Almost all the time | 5 (26.3) | 3 (15) | 0 (0) | 3 (15) | 2 (10) | 13 (13) |

**The bold values indicate the total number of respondents that replied to that question.**

**Supplementary table 3. Distribution of pig farmers responses regarding biosecurity measures within the farms, and cleaning and disinfection in selected districts of Uganda with high levels of suspect ASFV cases, June 2022 through July 2022.**

|  | **Number (%) of respondents** | | | | | |
| --- | --- | --- | --- | --- | --- | --- |
|  | **Mpigi** | **Masaka** | **Luwero** | **Kamuli** | **Wakiso** | **All districts** |
| **Total number of respondents per district** | 19 (100) | 20 (100) | 20 (100) | 20 (100) | 20 (100) | 99 (100) |
| **Biosecurity category: Measures within the farm/piggery** | | | | | | |
| **Washes hands before working with pigs** |  |  |  |  |  |  |
| No | 9 (47.4) | 6 (30) | 10 (50) | 8 (40) | 11 (55) | 44 (44.4) |
| Rarely | 0 (0) | 3 (15) | 1 (5) | 0 (0) | 0 (0) | 4 (4.0) |
| Sometimes | 5 (26.3) | 6 (30) | 5 (25) | 3 (15) | 3 (15) | 22 (22.2) |
| Almost all the time | 5 (26.2) | 5 (25) | 4 (20) | 9 (45) | 6 (30) | 29 (29.3) |
| **Uses clothes dedicated to working with pigs** |  |  |  |  |  |  |
| No | 14 (73.7) | 12 (60) | 14 (70) | 13 (65) | 11 (55) | 64 (65) |
| Rarely | 2 (10.5) | 2 (10) | 2 (10) | 1 (5) | 0 (0) | 7 (7) |
| Sometimes | 1 (5.3) | 1 (5) | 3 (15) | 2 (10) | 5 (25) | 12 (12) |
| Almost all the time | 2 (10.5) | 5 (25) | 1 (5) | 4 (20) | 4 (20) | 16 (16) |
| **Uses footwear dedicated to working with pigs** |  |  |  |  |  |  |
| No | 11 (57.9) | 6 (30) | 9 (45) | 8 (40) | 7 (35) | 41 (41.4) |
| Rarely | 3 (15.8) | 1 (5) | 0 (0) | 1 (5) | 1 (5) | 6 (6.1) |
| Sometimes | 3 (15.8) | 1 (5) | 3 (15) | 2 (10) | 3 (15) | 12 (12.1) |
| Almost all the time | 2 (10.5) | 12 (60) | 8 (40) | 9 (45) | 9 (45) | 40 (40.4) |
| **Biosecurity category: Cleaning and disinfection** | | | | | | |
| **Pig pen/holding area regularly cleaned** | 19 (100) | 20 (100) | 20 (100) | 20 (100) | 20 (100) | 99 (100) |
| **Use disinfectant for cleaning** | 1 (5.3) | 1 (5) | 0 (0) | 1 (5) | 3 (15) | 6 (6.1) |
| **Use bleach (sodium hypochlorite) as disinfectant** | 1 (100) | 1 (100) | 0 (0) | 0 (0) | 0 (0) | 2 (33.3) |

**Supplementary table 4. The distribution of the responses regarding the management of sick and dead pigs in selected districts of Uganda with high levels of suspect ASFV cases, June 2022 through July 2022.**

|  | **Number (%) of respondents** | | | | | |
| --- | --- | --- | --- | --- | --- | --- |
|  | **Mpigi** | **Masaka** | **Luwero** | **Kamuli** | **Wakiso** | **All districts** |
|  | **19 (100)** | **20 (100)** | **20 (100)** | **20 (100)** | **20 (100)** | **99 (100)** |
| **Biosecurity category: Management of sick pigs** | | | | | | |
| Isolates sick pigs | 10 (52.6) | 13 (65) | 11 (55) | 16 (80) | 14 (70) | 64 (64.6) |
| Treats sick pigs | 18 (94.7) | 16 (80) | 19 (95) | 18 (90) | 18 (90) | 89 (90) |
| Sells sick pigs | 4 (21.1) | 2 (10) | 7 (35) | 4 (20) | 4 (20) | 21 (21.2) |
| Sells/consumes pork from the sick pigs | 0 (0) | 0 (0) | 1 (5.0) | 1 (5.0) | 2 (10) | 4 (4) |
| **Biosecurity category: Management of dead pigs** | | | | | | |
| **Bury dead pigs** | **19 (100)** | **20 (100)** | **17 (100)** | **17 (100)** | **18 (100)** | **91 (100)** |
| No | 7 (36.8) | 5 (25) | 2 (11.8) | 7 (41.2) | 2 (11) | 23 (25.3) |
| Rarely | 2 (10.5) | 2 (10) | 0 (0) | 0 (0) | 1 (5.6) | 5 (5.5) |
| Sometimes | 1 (5.3) | 3 (15) | 2 (11.8) | 3 (17.6) | 3 (16.7) | 12 (13.2) |
| Almost all the time | 9 (47.4) | 10 (50) | 13 (76.5) | 7 (41.2) | 12 (66.7) | 51 (56) |
| **Burns dead pigs** | **19 (100)** | **20 (100)** | **17 (100)** | **17 (100)** | **18 (100)** | **91 (100)** |
| No | 18 (94.7) | 17 (85) | 16 (94.1) | 17 (100) | 17 (94.4) | 85 (93.4) |
| Rarely | 1 (5.3) | 1 (5) | 0 (0) | 0 (0) | 0 (0) | 2 (2.2) |
| Sometimes | 0 (0) | 2 (10) | 1 (5.9) | 0 (0) | 1 (5.6) | 4 (4.4) |
| Almost all the time | 0 (0) | 0 (0) | 0 (0) | 0 (0) | 0 (0) | 0 (0) |
| **Feed dead pigs to dogs** | **19 (100)** | **20 (100)** | **17 (100)** | **17 (100)** | **18 (100)** | **91 (100)** |
| No | 17 (89.5) | 17 (85) | 14 (82.3)) | 17 (100) | 17 (94.4) | 82 (90.1) |
| Rarely | 1 (5.3) | 1 (5) | 0 (0) | 0 (0) | 1 (5.6) | 3 (3.3) |
| Sometimes | 1 (5.3) | 2 (10) | 2 (11.8) | 0 (0) | 0 (0) | 5 (5.5) |
| Almost all the time | 0 (0) | 0 (0) | 1 (5.9) | 0 (0) | 0 (0) | 1 (1.1) |
| **Sell pork from dead pigs** | **19 (100)** | **20 (100)** | **17 (100)** | **17 (100)** | **18 (100)** | **91 (100)** |
| No | 18 (94.7) | 20 (100) | 16 (94.1) | 17 (100) | 18 (100) | 89 (97.8) |
| Rarely | 1 (5.3) | 0 (0) | 0 (0) | 0 (0) | 0 (0) | 1 (1.1) |
| Sometimes | 0 (0) | 0 (0) | 1 (5.9) | 0 (0) | 0 (0) | 1 (1.1) |
| Almost all the time | 0 (0) | 0 (0) | 0 (0) | 0 (0) | 0 (0) | 0 (0) |
| **Dead pigs thrown to the bushes** | **19 (100)** | **20 (100)** | **17 (100)** | **17 (100)** | **18 (100)** | **91 (100)** |
| No | 15 (78.9) | 17 (85) | 15 (88.2) | 15 (88.2) | 17 (94.4) | 79 (86.8) |
| Rarely | 1 (5.3) | 1 (5) | 0 (0) | 0 (0) | 0 (0) | 2 (2.2) |
| Sometimes | 2 (10.5) | 1 (5) | 2 (11.8) | 1 (5.9) | 1 (5.6) | 7 (7.7) |
| Almost all the time | 1 (5.3) | 1 (5) | 0 (0) | 1 (5.9) | 0 (0) | 3 (3.3) |

**The bold values indicate the total number of respondents that replied to that question.**

**Supplementary table 5: Distribution of pig farmers biosecurity practices based on field observations in Mpigi district of Uganda with high levels of suspect ASFV cases, June 2022.**

|  | **Number (%) of respondents** | | |
| --- | --- | --- | --- |
|  | **No** | **Yes** | **Not observed** |
| Human food leftovers observed in feeding troughs | 13 (68.4) | 6 (31.6) | 0 (0) |
| Meat scraps observed* | 3 (50) | 3 (50) | 0 (0) |
| Any wild pigs such as warthogs or bushpigs around the homestead or village | 9 (47.4) | 0 (0) | 10 (52.6) |
| *Ornithodoros* spp. ticks seen in the housing in which pigs are kept | 12 (63.2) | 0 (0) | 7 (36.8) |
| Hand washing facility near the area where pigs are kept | 16 (84.2) | 0 (0) | 3 (15.8) |
| Any farm specific clothes and footwear | 13 (68.4) | 1 (5.3) | 5 (26.3) |
| Pig pen/holding area appears regularly cleaned | 2 (10.5) | 17 (89.5) | 0 (0) |
| Farm equipment appears clean | 6 (31.6) | 1 (5.3) | 12 (63.1) |
| Flies and/or rodents around pigs | 2 (10.5) | 17 (89.5) | 0 (0) |
| Dogs and/or cats mingling or in contact with pigs | 8 (42.1) | 5 (26.3) | 6 (31.6) |
| Other livestock or poultry mingling or in contact with pigs | 3 (15.8) | 11 (57.9) | 5 (26.3) |
| Visitors have contact with the pigs | 1 (5.3) | 3 (15.8) | 15 (78.9) |
| Manure from pigs spread to nearby crop fields or gardens | 0 (0) | 14 (73.7) | 5 (26.3) |

**Supplementary table 6A: Distribution of pig farmers biosecurity practices based on field observations in Masaka, Luwero, Kamuli, and Wakiso districts of Uganda, June and July 2022.**

|  | **Number (%) of respondents** | | | | |
| --- | --- | --- | --- | --- | --- |
|  | **Masaka** | **Luwero** | **Kamuli** | **Wakiso** | **All 4 districts** |
| **Biosecurity items with binary outcome variable** | | | | | |
| **Number of farm/piggery observations** | **20** | **20** | **20** | **20** | **80** |
| Chain link fence or wall on the property | 8 (40) | 2 (10) | 6 (30) | 3 (15) | 19 (23.8) |
| **Number of farm/piggery observations** | **8** | **2** | **6** | **3** | **19** |
| Fence surrounds entire property | 8 (100) | 2 (100) | 5 (83.3) | 2 (66.7) | 17 (89.5) |
| **Number of farm/piggery observations** | **19** | **20** | **20** | **20** | **79** |
| Human food leftovers observed in feeding troughs | 3 (15.8) | 4 (20) | 0 (0) | 2 (10) | 9 (11.4) |
| **Number of farm/piggery observations** | **3** | **4** | **0** | **2** | **9** |
| Meat scraps observed | 0 (0) | 1 (25) | 0 (0) | 0 (0) | 1 (11.1) |
| **Number of farm/piggery observations** | **20** | **20** | **20** | **20** | **80** |
| *Ornithodoros* spp. ticks seen in the housing in which pigs are kept | 0 (0) | 0 (0) | 0 (0) | 0 (0) | 0 (0) |
| **Number of farm/piggery observations** | **19** | **20** | **20** | **20** | **79** |
| Hand washing facility near the area where pigs are kept | 6 (31.6) | 0 (0) | 0 (0) | 1 (5) | 7 (8.9) |
| **Number of farm/piggery observations** | **19** | **20** | **20** | **20** | **79** |
| Farm specific clothing seen near the pig pens or pig keeping area | 1 (5.3) | 0 (0) | 0 (0) | 0 (0) | 1 (1.3) |
| **Number of farm/piggery observations** | **19** | **20** | **20** | **20** | **79** |
| Footwear such as boots that appear to be dedicated to work on pigs seen near the pig keeping area | 0 (0) | 0 (0) | 0 (0) | 0 (0) | 0 (0) |
| **Number of farm/piggery observations** | **20** | **20** | **20** | **20** | **80** |
| Pig pen/holding area appear regularly cleaned | 19 (95) | 11 (55) | 13 (65) | 10 (50) | 53 (66.3) |
| **Number of farm/piggery observations** | **19** | **20** | **20** | **20** | **79** |
| Enclosure or area that could be dedicated for quarantine of new pigs observed | 10 (52.6) | 6 (30) | 9 (45) | 8 (40) | 33 (41.8) |
| **Number of farm/piggery observations** | **19** | **20** | **20** | **20** | **79** |
| There is evidence of fly and/or rodent control | 5 (26.3) | 5 (20) | 0 (0) | 0 (0) | 9 (11.4) |
| **Number of farm/piggery observations** | **20** | **20** | **20** | **20** | **80** |
| There is potential for dogs and/or cats mingling or coming in contact with pigs | 9 (45) | 13 (65) | 12 (60) | 15 (75) | 49 (61.5) |
| **Number of farm/piggery observations** | **20** | **20** | **20** | **20** | **80** |
| There potential for poultry mingling or coming in contact with pigs | 14 (70) | 15 (75) | 16 (80) | 18 (90) | 63 (78.8) |
| **Number of farm/piggery observations** | **20** | **20** | **20** | **20** | **80** |
| There potential for other livestock mingling or coming in contact with pigs | 2 (10) | 9 (45) | 8 (40) | 9 (45) | 28 (35) |

**The bold values indicate the total number of respondents that replied to that question.**

**Supplementary table 6B: Distribution of pig farmers biosecurity practices based on the field observations in Masaka, Luwero, Kamuli, and Wakiso districts of Uganda, June 2022.**

|  | **Number (%) of respondents** | | | | |
| --- | --- | --- | --- | --- | --- |
|  | **Masaka** | **Luwero** | **Kamuli** | **Wakiso** | **All 4 districts** |
| **Biosecurity items with more than two response categories** |  | | | | |
| **Visitors have contact with the pigs** | **20 (100)** | **20 (100)** | **20 (100)** | **20 (100)** | **80 (100)** |
| No | 2 (10) | 1 (5) | 2 (10) | 2 (10) | 7 (8.8) |
| Yes | 1 (5) | 1 (5) | 1 (5) | 0 (0) | 3 (3.8) |
| Not observed | 17 (85) | 18 (90) | 17 (85) | 18 (90) | 70 (80.5) |
| **Proximity of the pigs to the living area** | **20 (100)** | **20 (100)** | **20 (100)** | **20 (100)** | **80 (100)** |
| Designated pig area very close to the living area | 8 (40) | 6 (30) | 6 (30) | 7 (35) | 27 (33.8) |
| Designated pig area is on the property but semi-removed from the living area | 9 (45) | 10 (50) | 11 (55) | 7 (35) | 37 (46.2) |
| Designated pig area is far from way from the living area | 3 (15) | 4 (20) | 3 (15) | 6 (30) | 16 (20) |
| **Manure from pigs spread to nearby crop fields or gardens** | **20 (100)** | **20 (100)** | **20 (100)** | **20 (100)** | **80 (100)** |
| No | 1 (5) | 0 (0) | 1 (5) | 1 (5) | 3 (7.8) |
| Yes | 10 (50) | 12 (60) | 9 (45) | 5 (25) | 36 (45) |
| Not observed | 9 (45) | 8 (40) | 10 (50) | 14 (70) | 41 (51.2) |

**The bold values indicate the total number of respondents that replied to that question.**

**Supplementary table 7. Characteristics of the key informants in a survey of biosecurity practices of pig farmers in selected districts of Uganda with high levels of suspect ASFV cases, June 2022 through July 2022.**

| **Characteristics of Key informants** | |
| --- | --- |
| **Key informants area of work (n = 19)** | **Number (percentage)** |
| Local government | 16 (84.2) |
| Ministry of Agriculture, Animal Industry and Fisheries | 1 (5.3) |
| Academia | 1 (5.3) |
| Private sector | 1 (5.3) |
| **District where workplace is located (n = 16)** |  |
| Mpigi | 0 (0) |
| Masaka | 2 (12.5) |
| Luwero | 1 (6.2) |
| Kamuli | 0 (0) |
| Wakiso | 13 (81.3) |

**Supplementary table 8A. A summary of the key informants’ opinions regarding pig farmers biosecurity practices in selected districts of Uganda with high levels of suspect ASFV cases, June 2022 through July 2022.**

|  | **Number (%) of respondents** | | | | | |
| --- | --- | --- | --- | --- | --- | --- |
| **Category of response chosen** | **None** | **1-25%** | **26-50%** | **51-75%** | **76-100%** | **Didn’t know** |
| Farmers that have a fence (n = 19) | 0 (0) | 10 (52.6) | 7 (36.8) | 1 (5.3) | 1 (5.3) | 0 (0) |
| Farmers that quarantine new animals before their introduction to other pigs (n = 19) | 3 (15.8) | 12 (63.2) | 1 (5.3) | 1 (5.3) | 2 (10.5) | 0 (0) |
| Farmers that evaluate their pigs before taking them out of quarantine (n =16) | 3 (18.7) | 9 (56.2) | 2 (12.5) | 1 (6.3) | 1 (6.3) | 0 (0) |
| Farmers that return unsold animals to their farms/homesteads from the market and keep them with other pigs (n = 19) | 5 (26.3) | 4 (21) | 1 (5.3) | 1 (5.3) | 5 (26.3) | 3 (15.8) |
| Farmers that use household leftovers, restaurant waste, leftovers from parties or other social functions that contain meat scraps as pig food (n = 19) | 0 (0) | 3 (15.8) | 4 (21) | 6 (31.6) | 6 (31.6) | 0 (0) |
| Farmers that cook or boil the waste before feeding it to pigs (n =18) | 5 (27.8) | 7 (38.9) | 4 (22.2) | 1 (5.6) | 1 (5.6) | 0 (0) |
| Farmers that routinely provide foot baths with disinfectant at the entrance to their pig housing (n = 18) | 0 (0) | 14 (77.8) | 2 (11.1) | 2 (11.1) | 0 (0) | 0 (0) |
| Farmers that let people who have contact with pigs from other farms/households contact their pigs (n = 18) | 0 (0) | 0 (0) | 5 (27.8) | 10 (55.6) | 2 (11.1) | 1 (5.6) |
| Farmers that provide visitors with farm specific clothes and/or footwear when they contact pigs (n = 18) | 8 (44.4) | 9 (50) | 0 (0) | 0 (0) | 1 (5.6) | 0 (0) |
| Where footwear is not provided, proportion of visitors that clean their footwear before contacting the pigs (n = 8) | 3 (37.5) | 4 (50) | 1 (12.5) | 0 (0) | 0 (0) | 0 (0) |
| Farmers that control flies around their pigs (n = 18) | 4 (22.2) | 8 (44.4) | 1 (5.6) | 3 (16.7) | 1 (5.6) | 1 (5.6) |
| Farmers control rodents around their pigs (n = 18) | 5 (27.8) | 10 (55.6) | 1 (5.6) | 1 (5.6) | 1 (5.6) | 0 (0) |
| Farms where household pigs have contact with village dogs (n = 18) | 0 (0) | 3 (16.7) | 3 (16.7) | 4 (22.2) | 6 (33.3) | 2 (11.1) |
| Percentage of household pigs that have contact with cats (n = 18) | 0 (0) | 4 (22.2) | 4 (22.2) | 2 (11.1) | 5 (27.8) | 3 (16.7) |
| Percentage of farmers have pigs that come in contact with other livestock (n = 18) | 0 (0) | 6 (33.3) | 4 (22.2) | 4 (22.2) | 3 (16.7) | 1 (5.6) |
| Percentage of farmers have pigs that come in contact with poultry (n = 17) | 0 (0) | 5 (29.4) | 2 (11.8) | 5 (29.4) | 5 (29.4) | 0 (0) |
| Percentage of farmers have pigs that come in regular contact with pigs of other farmers (n = 18) | 3 (16.7) | 8 (44.4) | 4 (22.2) | 1 (5.6) | 1 (5.6) | 1 (5.6) |
| Percentage of pig farmers report that wild pigs, such as warthogs or bush pigs, roam around their homesteads or villages (n =18) | 14 (77.8) | 2 (11.1) | 0 (00 | 0 (0) | 0 (0) | 2 (11.1) |
| Percentage of farmers report that wild pigs come into contact with their pigs (n = 4) | 0 (0) | 2 (50) | 0 (0) | 0 (0) | 0 (0) | 2 (50) |
| Percentage of pig farmers that clean their pig pens or holding area (n = 18) | 0 (0) | 1 (5.5) | 3 (16.7) | 7 (38.9) | 7 (38.9) | 0 (0) |
| Farmers that commonly share equipment with other pig farmers (n = 18) | 5 (27.8) | 7 (38.9) | 4 (22.2) | 0 (0) | 0 (0) | 2 (11.1) |
| Farmers that clean and disinfect equipment between farms (n = 11) | 2 (18.2) | 6 (54.5) | 2 (18.2) | 0 (0) | 1 (9.1) | 0 (0) |

**Supplementary table 8B. A summary of the key informants’ opinions regarding pig farmers biosecurity practices in selected districts of Uganda with high levels of suspect ASFV cases, June 2022 through July 2022**

|  | **Number (%) of respondents** | | | | |  |  |
| --- | --- | --- | --- | --- | --- | --- | --- |
|  | **Not common** | | **Common** | **Very common** | **Didn’t know** |  |  |
| How common is it for farmers to involve animal health workers in evaluating the health status of pigs brought to their farms? (n = 18) | 8 (44.4) | | 8 (44.4) | 2 (11.1) | 0 (0) |  |  |
| How common it is for pig farmers to use household waste as feed for their pigs? (n = 19) | 0 (0) | | 6 (31.6) | 13 (68.4) | 0 (0) |  |  |
| How common it is for pig farmers to use restaurant waste as feed for their pigs? (n = 18) | 0 (0) | | 12 (66.7) | 6 (33.3) | 0 (0) |  |  |
| Is it common for farmers to isolate sick pigs from healthy ones? (n = 18) | 6 (33.3) | | 9 (50) | 2 (11.1) | 1 (5.6) |  |  |
| Is it common for farmers to treat sick pigs? (n = 18) | 1 (5.6) | | 9 (50) | 7 (38.9) | 1 (5.6) |  |  |
| Is it common for farmers to sell off sick pigs? (n = 18) | 0 (0) | | 8 (44.4) | 9 (50) | 1 (5.6) |  |  |
| Is it common for farmers to slaughter and consume pork from sick pigs? (n = 17) | 3 (17.6) | | 12 (70.6) | 2 (11.8) | 0 (0) |  |  |
| Is it common for farmers to slaughter and sell pork from sick pigs? (n = 18) | 3 (16.7) | | 9 (50) | 4 (22.2) | 2 (11.1) |  |  |
| Is it common for farmers to wash their hands before working with pigs? (n = 18) | 15 (83.3) | | 3 (16.7) | 0 (0) | 0 (0) |  |  |
| How common is it for farmers to put on clean clothes before working with pigs? (n = 18) | 16 (88.9) | | 1 (5.6) | 0 (0) | 1 (5.6) |  |  |
| How common is it for farmers to put on clean footwear before working with pigs? (n = 18) | 12 (66.7) | | 5 (27.8) | 0 (0) | 1 (5.6) |  |  |
| **How common is it for pig farmers to allow these categories of visitors to go into the areas where pigs are kept?** | **Never allowed** | **Least commonly allowed** | | **Commonly allowed** | **Most commonly allowed** | **Didn’t know** |  |
| Neighbors (n = 18) | 0 (0) | 4 (22.2) | | 8 (44.4) | 5 (27.8) | 1 (5.6) |  |
| Animal health workers (n = 18) | 0 (0) | 0 (0) | | 5 (27.8) | 13 (72.2) | 0 (0) |  |
| Community leaders (n = 18) | 0 (0) | 5 (27.8) | | 9 (50) | 3 (16.7) | 1 (5.6) |  |
| Family (n = 17) | 0 (0) | 0 (0) | | 4 (23.5) | 13 (76.5) | 0 (0) |  |
| Pig buyers (n = 18) | 0 (0) | 2 (11.1) | | 7 (38.9) | 9 (50) | 0 (0) |  |
| **How common are the following practices as methods of disposing dead pigs by farmers?** | **Not at all common** | **Slightly common** | | **Moderately common** | **Very common** | **Extremely common** | **Didn’t know** |
| Buried (n = 17) | 2 (11.8) | 5 (29.4) | | 4 (23.5) | 5 (29.4) | 1 (5.9) | 0 (0) |
| Burned (n = 18) | 13 (72.2) | 4 (22.2) | | 1 (5.6) | 0 (0) | 0 (0) | 0 (0) |
| Fed to dogs (n =18) | 1 (5.6) | 11 (61.1) | | 2 (11.1) | 3 (16.7) | 0 (0) | 1 (5.6) |
| Pork from dead pigs eaten at home (n = 18) | 6 (33.3) | 5 (27.8) | | 2 (11.1) | 1 (5.6) | 1 (5.6) | 3 (16.7) |
| Pork from dead pigs sold (n =18) | 3 (16.7) | 7 (38.9) | | 1 (5.6) | 6 (33.3) | 1 (5.6) | 0 (0) |
| Dead pigs thrown to bushes (n = 17) | 6 (35.3) | 3 (17.6) | | 6 (35.3) | 0 (0) | 0 (0) | 2 (11.8) |
